# Bioactivity and miRNome Profiling of Native Extracellular Vesicles in Human Induced Pluripotent Stem Cell‐Cardiomyocyte Differentiation

**DOI:** 10.1002/advs.202104296

**Published:** 2022-03-24

**Authors:** Ana F. Louro, Marta A. Paiva, Marta R. Oliveira, Katharina A. Kasper, Paula M. Alves, Patrícia Gomes‐Alves, Margarida Serra

**Affiliations:** ^1^ iBET Instituto de Biologia Experimental e Tecnológica Apartado 12 Oeiras 2781‐901 Portugal; ^2^ ITQB‐NOVA Instituto de Tecnologia Química e Biológica António Xavier Universidade Nova de Lisboa Av. da República Oeiras 2780‐157 Portugal

**Keywords:** cardiac regeneration, cell biofactory, extracellular vesicles, miRNome, small RNA‐seq

## Abstract

Extracellular vesicles (EV) are an attractive therapy to boost cardiac regeneration. Nevertheless, identification of native EV and corresponding cell platform(s) suitable for therapeutic application, is still a challenge. Here, EV are isolated from key stages of the human induced pluripotent stem cell‐cardiomyocyte (hiPSC‐CM) differentiation and maturation, i.e., from hiPSC (hiPSC‐EV), cardiac progenitors, immature and mature cardiomyocytes, with the aim of identifying a promising cell biofactory for EV production, and pinpoint the genetic signatures of bioactive EV. EV secreted by hiPSC and cardiac derivatives show a typical size distribution profile and the expression of specific EV markers. Bioactivity assays show increased tube formation and migration in HUVEC treated with hiPSC‐EV compared to EV from committed cell populations. hiPSC‐EV also significantly increase cell cycle activity of hiPSC‐CM. Global miRNA expression profiles, obtained by small RNA‐seq analysis, corroborate an EV‐miRNA pattern indicative of stem cell to cardiomyocyte specification, confirming that hiPSC‐EV are enriched in pluripotency‐associated miRNA with higher in vitro pro‐angiogenic and pro‐proliferative properties. In particular, a stemness maintenance miRNA cluster upregulated in hiPSC‐EV targets the PTEN/PI3K/AKT pathway, involved in cell proliferation and survival. Overall, the findings validate hiPSC as cell biofactories for EV production for cardiac regenerative applications.

## Graphical abstract

Extracellular vesicles (EV) from key stages of the hiPSC‐cardiomyocyte differentiation and maturation are characterized, and EV bioactivity, particularly for native hiPSC‐EV, is shown. These EV are enriched in stemness maintenance miRNA, predicted to target the PI3K/AKT pathway, intrinsically involved in cell survival and proliferation. The potential of hiPSC as cell biofactories for the production of therapeutic EV is validated.

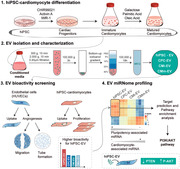

## 1 Introduction

EV are a diverse population of membrane enclosed nano‐ and microparticles naturally released by cells not only during normal cellular physiology, but also during stress and disease.^[^ ^1^ ^]^ They contain and transport several bioactive molecules such as proteins, nucleotides, lipids and metabolites, and are thought to specifically deliver their cargo to target cells, possibly through the presence of distinctive surface markers.^[^ ^2^, ^3^ ^]^ In eukaryotic cells, EV secretion occurs through shedding of the plasma membrane, fusion of multivesicular bodies with the plasma membrane, or during controlled cell death. Hence, EV are classified according to their origin and size, being described as microvesicles (100–1000 nm), exosomes (30–200 nm), or apoptotic bodies (1–5 µm).^[^ ^3^, ^4^, ^5^ ^]^

In cardiac regenerative medicine, there is a growing interest in using EV as cell‐mimetic therapeutics, not only due to their promising therapeutic superiority, but also due to their advantages over cell transplantation, such as absence of oncologic risk, low immunogenicity, possibility of large‐scale production, off‐the‐shelf storage, and availability.^[^ ^6^, ^7^ ^]^ Several studies have focused on the cardioprotective and angiogenic potential of EV‐derived from stem cells,^[^ ^8^, ^9^ ^]^ cardiac progenitors,^[^ ^10^, ^11^, ^12^, ^13^ ^]^ and cardiomyocytes.^[^ ^14^ ^]^ Recently, EV were shown to be virtually non‐immunogenic, crossing an important prerequisite for their clinical translation.^[^ ^7^, ^15^ ^]^ Mechanistically, EV activity is still unclear. EV‐mediated horizontal transfer of genetic material has been shown to trigger effects on recipient cells and to regulate protein expression,^[^ ^16^, ^17^ ^]^ with EV‐carried microRNA (miRNA) considered to be an important active cargo modulating this intercellular communication.^[^ ^16^, ^18^ ^]^

miRNA are small strands (≈22 nucleotides) of non‐coding RNA that bind to the 3′‐UTR seed region of target mRNA sequences, inhibiting its translation or inducing its cleavage, according to the degree of complementarity. miRNA participate in crucial biological processes, and have been associated with cell proliferation, differentiation, tissue development events, and apoptosis.^[^ ^19^ ^]^ Not all miRNAs are ubiquitously expressed, with tissue‐specific subsets,^[^ ^20^ ^]^ such as myomiRs (miR‐1, miR‐133a, miR‐133b, miR‐206, miR‐208a, miR‐208b, miR‐486, and miR‐499), exclusively or preferentially expressed in striated muscle,^[^ ^21^, ^22^, ^23^, ^24^, ^25^ ^]^ and involved in muscle cell proliferation and/or differentiation, or angiomiRs (miR‐126, miR‐130a, let‐7f, and the miR‐17‐92 cluster), expressed in endothelial cells and identified as key regulators in angiogenic processes.^[^ ^26^, ^27^, ^28^, ^29^, ^30^ ^]^ Similarly, miRNA clusters were shown to be essential in stem cell maintenance (miR‐106a‐363,^[^ ^31^ ^]^ miR‐302a‐367,^[^ ^32^, ^33^ ^]^ miR‐17‐92,^[^ ^31^ ^]^ and C19MC clusters,^[^ ^34^ ^]^ and miR‐200c/b)^[^ ^35^, ^36^ ^]^ and differentiation.^[^ ^31^, ^37^ ^]^ In fact, combinations of miRNAs were capable of inducing direct cellular reprogramming (reviewed in refs. [38, 39]), either back to a pluripotent state, or producing phenotype switches relevant in a cardiac repair context.^[^ ^40^ ^]^

The adult mammalian heart is a highly structured organ composed of cardiomyocytes, fibroblasts, mural, endothelial, and immune cells.^[^ ^41^ ^]^ Inter‐ and intra‐cellular crosstalk are vital during cardiac development, in normal cardiac function, and in pathological conditions. In a healthy myocardium, cardiomyocytes are surrounded by an intricate network of capillaries, which provide nutrients, an oxygenated blood source, and signals that promote cardiomyocyte organization and survival.^[^ ^41^ ^]^ In the occurrence of an acute myocardial infarction typically there is an extensive and permanent myocyte loss due to the disruption of this network. To promote myocardial regeneration EV should be able to enhance endogenous repair pathways, either through stimulation of cell proliferation, activation of resident progenitor cells, induction of angiogenesis, reduction of fibrosis, or by triggering a reparative immune response in injured hearts.^[^ ^42^, ^43^ ^]^ The relative importance of targeting one process versus the other will depend on the clinical context, considering whether there is a predominance of ischemia or ventricular dysfunction and scarring.

Studies on the cardiac repair effect of EV traditionally use mesenchymal or cardiac progenitor cell‐derived EV, overlooking the potential of human induced pluripotent stem cells (hiPSC) and hiPSC‐derived cardiomyocytes (hiPSC‐CM) as alternative EV sources. Hence, we sought to characterize the EV(s) and corresponding cell platform(s) suitable for cardiac therapeutic applications along hiPSC‐CM differentiation, and to identify genetic signatures of therapeutically relevant EV. To achieve this, we differentiated cardiomyocytes from hiPSC, promoted their metabolic maturation,^[^ ^44^, ^45^ ^]^ and separated EV from conditioned media at the hiPSC, cardiac progenitor cell (CPC), immature (CMi), and more mature (CMm) cardiomyocyte stages. We then compared their functionality and characterized their miRNome. We concluded that hiPSC‐EV, enriched in pluripotency‐associated miRNAs, show higher in vitro pro‐angiogenic and pro‐proliferative properties in comparison with EV derived from partially and terminally committed cells.

## 2 Results

### 2.1 EV Are Secreted Along Differentiation and Maturation of hiPSC into Cardiomyocytes

**Figure 1:**
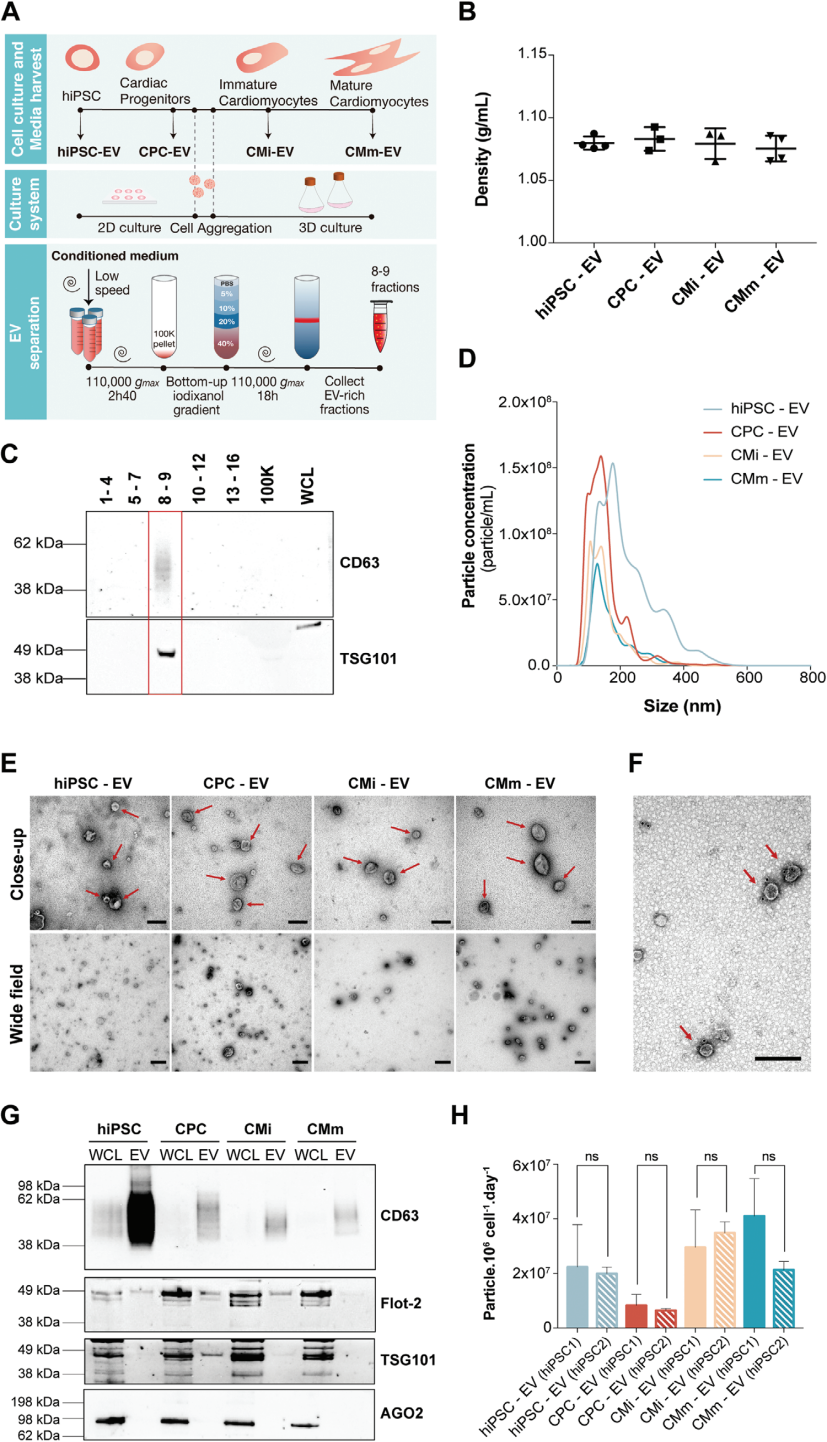
Schematic representation of the experimental design and EV separation method validation. A) Overview of the cardiomyocyte differentiation and EV isolation processes from the different cell populations—human induced pluripotent stem cells (hiPSC) and their derivatives including cardiac progenitor cells (CPC) and cardiomyocytes at different stages of maturation (immature (CMi) and more mature (CMm) cells). B) Density of EV isolated in 8–9 gradient fractions. C) Validation of the EV separation method by western blot analysis of CD63 and TSG101 on density gradient fractions after ultracentrifugation. 100K—pellet resulting from the first ultracentrifugation. WCL: whole cell lysate. D) Typical size distribution profile of EV samples analyzed by nanoparticle tracking analysis (NTA). Plotted lines correspond to the averaged size distribution profiles from three EV isolations. E) Representative negative staining close‐up and wide‐field transmission electron microscopy (TEM) images of all EV samples. EVs are marked by red arrows. Scale bars: close‐up: 200 nm; wide‐field: 500 nm. F) Representative CD63 immunogold labeling of hiPSC‐EV. CD63‐labeled EVs are marked by red arrows. Scale bar: 200 nm. G) Western blot analysis of common EV markers (CD63, Flotilin‐2, and TSG101) and co‐isolated contaminants (AGO2) for 8–9 pooled gradient fractions. WCL: whole cell lysate. H) EVs yield obtained along hiPSC‐CM differentiation and maturation for both cell lines studied. No significant differences were found between cell lines, for each differentiation stage. In (B) and (H) results are plotted as mean ± SD (*n* = 3). n.s. (*p* > 0.05) by one‐way ANOVA with Sidak's multiple comparisons test, with a single pooled variance.

hiPSC were cultured in 2D monolayers and differentiated toward hiPSC‐CM using the protocol previously published by our group.^[^ ^45^ ^]^ Beating cells were typically observed on day 7 after differentiation was initiated, at which point they were aggregated and transferred to a 3D dynamic culture system and maintained throughout the experiment (**Figure** **1**A). Cardiomyocyte maturation was promoted both by providing a 3D culture environment and enforcing a metabolic switch, achieved by culturing cardiomyocytes in a glucose depleted medium supplemented with galactose and fatty‐acids, thereby improving structural, functional, and metabolic maturation, and providing a cellular architecture with higher biological relevance for EV production.^[^ ^44^, ^45^, ^46^, ^47^ ^]^ Cells were characterized at each CondM collection time‐point to ensure their phenotypic and genotypic identity (Figure S1, Supporting Information). hiPSC were positive for pluripotency markers SSEA‐4 and TRA‐1‐60, and negative for early differentiation marker SSEA‐1 (Figure S1A, Supporting Information). CPC identity was confirmed by increased expression of *GATA4* and *ISL1*, genes implicated in cardiac commitment and promotion of cardiogenesis (Figure S1B,C, Supporting Information). CMi were positive for cardiomyocyte‐specific markers cardiac troponin T (cTnT > 80%), SIRPa/b and VCAM‐1 (Figure S1D, Supporting Information). CMm were over 85% positive for cTnT (Figure S1E, Supporting Information) and were additionally characterized by positive immunofluorescence stainings for cTnT and connexin 43 (Figure S1H,I, Supporting Information). Cardiomyocytes at day 35 of culture showed increased ratios of adult to fetal isoforms of sarcomeric genes when compared to day 15, including *MYH7/MYH6* and *TNNI3/TNNI1*, encoding adult/fetal myosin heavy chain and cardiac/slow skeletal muscle troponin I, respectively (Figure S1F,G, Supporting Information). These results confirm a distinct maturation profile for CMm.^[^ ^44^ ^]^

Throughout the culture period, cell viability was monitored, in order to diminish the potential contribution of apoptotic bodies to the small‐EV enriched preparation. Viability was estimated to be higher than 85% at all CondM collection points (Figure S1J,K, Supporting Information). Cell counting with Trypan Blue exclusion dye was not performed for spheroids due to the need of aggressive dissociation techniques that would interfere with cell viability.

CondM was harvested at days 0 (hiPSC; expansion medium), 6 (CPC; differentiation medium), 15 (CMi; maintenance medium) and 35 (CMm; maturation medium) of the hiPSC‐CM differentiation (Figure 1A). EV were separated from CondM using an OptiPrep density gradient ultracentrifugation (ODG) (Figure 1A). Pooled fractions 8 and 9 from the ODG corresponded to a buoyant density of ≈1.08 g mL^−1^ and were positive for EV‐associated markers CD63 and TSG101 (Figure 1B,C and Figure S2A, Supporting Information), thus validating the efficiency of ODG in separating EV from CondM.

Standard particle characterization was conducted for EV‐rich 8–9 fractions from each parent cell. Nanoparticle tracking analysis (NTA) indicated a typical EV size distribution profile for all samples, with most particles occurring in the range of 50–250 nm of diameter (Figure 1D). Transmission electron microscopy (TEM) analysis confirmed the presence of vesicles with cup‐shaped morphology and absence of similar‐density contaminant particles (Figure 1E,F). To further demonstrate the identity of isolated EV samples, immunoblotting was performed for detection of specific markers CD63, TSG101, and Flotilin‐2, and absence of common co‐isolated contaminants (Argonaute‐2) (Figure 1G; Figure S2B, Supporting Information). In addition, the EV yield (estimated by the number of particles secreted per million cell per day) was obtained for each producing cell, having achieved the lowest value for CPC and the highest values for cardiomyocytes (CMi and CMm), consistently for both hiPSC lines studied (hiPSC1 and hiPSC2) (Figure 1H).

A similar characterization was performed for 8–9 fractions of gradients performed with 100k pellets of non‐CondM, to provide a portrayal of the background particles present in the four different culture media used (Figure S3, Supporting Information). Particles were detected by NTA in a significant smaller scale in comparison to CondM and no EV‐like particles were observed in TEM images (Figure S3A, Supporting Information). In fact, medium particles contributed to less than 5% of total particles isolated from CondM (Figure S3B, Supporting Information) and thus, no correction for background was performed in subsequent assays.

### 2.2 EV Are Uptaken by Cardiac and Endothelial Cells

**Figure 2:**
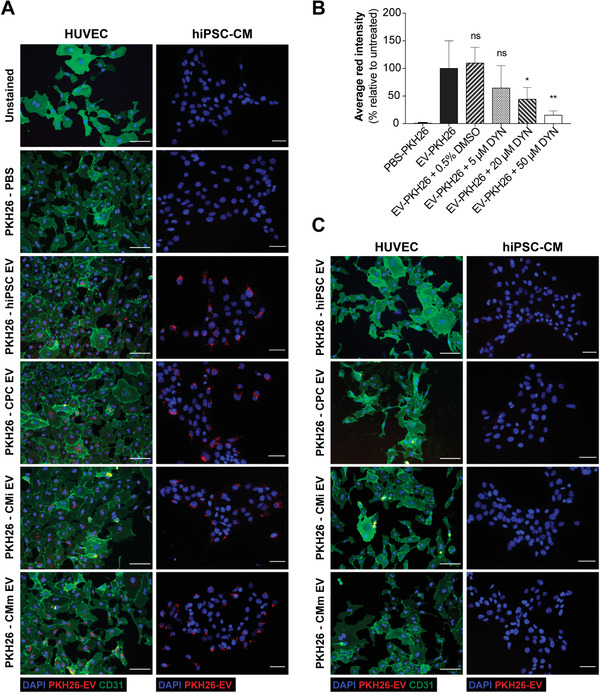
EV are uptaken by endothelial cells and cardiomyocytes. A) Representative immunofluorescence images of the uptake assays performed in HUVEC and hiPSC‐CM. B) Effects of Dynasore treatment on EV uptake. Quantification of the average red channel intensity corresponding to the emission range of PKH26 in HUVEC treated with PKH26‐EV in the absence or presence of increasing concentrations of Dynasore. Data presented as mean ± SD, *n* = 3, *p < 0.05, **p < 0.01, ns: nonsignificant versus EV‐PKH26 group by one‐way ANOVA with Dunnett's multiple comparisons test, with a single pooled variance. C) Representative immunofluorescence images of the uptake inhibition assay performed in HUVEC and hiPSC‐CM upon addition of 50 × 10^−6^ m of Dynasore. HUVECs were stained for the transmembrane protein CD31 (green), EV were labeled with PKH26 and nuclei were counterstained with DAPI (blue). Cells were observed under an inverted fluorescence microscope (DMI6000, Leica Microsystems GmbH, Germany). Scale bar: 100 µm.

To determine if EV from the four distinct parent cells were biologically active, we confirmed their uptake by endothelial cells (HUVEC) and hiPSC‐CM. PKH26 was used to label lipid rich EV membranes and detect their internalization. To discard the possibility of non‐EV nanoparticle formation from fluorescent dye, a PKH26‐labeled PBS control was prepared and subjected to the same isolation process as EV samples. Fluorescence images revealed all EV groups were present within cells, particularly in the peri‐nuclear region, with no apparent tropism toward a particular cell type (**Figure** **2**A). Uptake blockage was performed with the use of Dynasore, a GTPase inhibitor which acts on Dynamin‐2 to prevent detachment of the endocytic vesicle from the cell membrane during Caveolin‐dependent endocytosis/Clathrin‐mediated endocytosis and therefore inhibit EV internalization by recipient cells. The addition of Dynasore blocked EV uptake in a dose‐dependent manner (Figure 2B) and reduced the uptake of all EV groups (Figure 2C), as previously demonstrated.^[^ ^14^, ^48^ ^]^

### 2.3 hiPSC‐EV Have Greater In Vitro Pro‐Angiogenic and Pro‐Proliferative Activity than EV from Cardiac Committed Cell Populations

To assess and compare the therapeutic potential of EV derived from the four parent cell populations, we tested their ability to induce angiogenesis in vascular cells (HUVEC), and proliferation in hiPSC‐CM, as our interest was to target heart regeneration based on myocardial regrowth, supported by vascularization.

**Figure 3:**
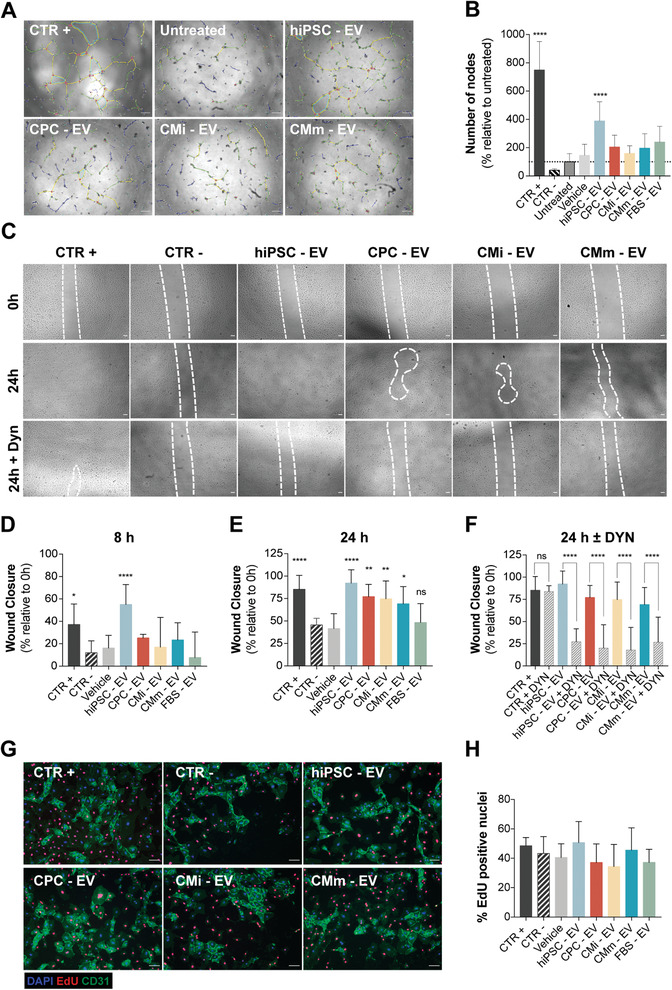
hiPSC‐EV promote angiogenesis and migration in HUVEC. A) Angiogenic potential of EV samples, evaluated as tube formation at 8 h post‐seeding. The number of nodes (pink dots), master junctions (pink circles), master segments (yellow), meshes (light blue), branches (green), and isolated segments (blue) are shown. Scale bar: 100 µm. B) Tube formation measured as percentage of number of nodes formed in the assay, relative to the untreated control (taken as 100%). Fully supplemented medium was used as positive control, 5 × 10^−6^ m Suramin Sodium Salt prepared in basal medium as negative control, basal medium as untreated control, and 8–9 fractions of a blank gradient as vehicle control. C) Effect of EV treatment on HUVEC migration evaluated by the wound healing assay. Representative images of cell migration at 0 and 24 h post‐scratch, with and without EV uptake inhibition by Dynasore (50 × 10^−6^ m). Scale bar: 100 µm. Wound closure at D) 8 h and E) 24 h post‐scratch. F) Wound closure at 24 h, with and without EV‐uptake inhibition, mediated by Dynasore (50 × 10^−6^ m). Wound closure measured as a percentage of the initial wound area. Fully supplemented medium was used as positive control, basal medium supplemented with 0.5% fetal calf serum minus growth factors, as negative control and 8–9 fractions of a blank gradient as vehicle control. G) HUVEC proliferation in the wound healing assay, assessed by EdU incorporation (red). HUVEC were stained for the transmembrane protein CD31 (green) and nuclei were counterstained with DAPI (blue). Purple nuclei in merged images correspond to proliferating cells. Scale bar: 100 µm. H) Quantification of EdU‐positive cells from five randomly selected fields per well, equivalent to a minimum of 1000 DAPI‐stained nuclei per experiment. No significant differences were observed for any of the samples. In (B), (D), (E), (F), and (H), results are plotted as mean ± SD (*n* = 3). In (B), (D), (E), and (H), significance was tested against the negative control. *p < 0.05, **p < 0.01, ****p < 0.0001, n.s. (*p* > 0.05) by one‐way ANOVA with Dunnett's multiple comparisons test, with a single pooled variance. In (F), significance was tested by one‐way ANOVA with Sidak's multiple comparisons test, with a single pooled variance. ****p < 0.0001, n.s. (*p* > 0.05). CTR+: positive control, CTR−: negative control.

The in vitro pro‐angiogenic effect of EV was evaluated through tube formation and wound healing assays. We observed that addition of EV to HUVEC resulted in the formation of longer segments and tubules, and stimulated network formation (**Figure** **3**A), as seen by the increase in the number of nodes when compared to basal medium alone (untreated group) (Figure 3B). However, a significant effect was only observed for hiPSC‐EV. EV treatment also increased cell migration, measured by the percentage of closure of a scratch created on an endothelial cell monolayer (Figure 3C). Overall, all EV groups stimulated wound closure; nevertheless, this effect was faster and to a greater extent in the hiPSC‐EV treated group (Figure 3D,E). To confirm that the observed effects were due to the presence of EV, we included a Dynasore‐treated group. While Dynasore did not hinder wound closure in the positive control, it significantly reduced migration in EV‐treated groups, thus confirming EV activity (Figure 3F). HUVECs’ cell cycle activity was monitored by EdU incorporation throughout the wound healing assays, to confirm wound closure was not due to different cell proliferation rates (Figure 3G,H). It is important to note that although the same trend was observed for hiPSC‐EV from both cell lines studied, EV from hiPSC1 showed a more pronounced effect (Figure S4, Supporting Information).

**Figure 4:**
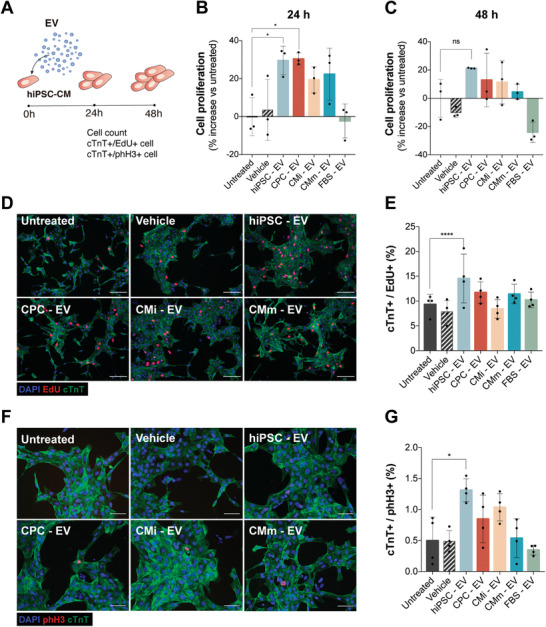
hiPSC‐EV promote hiPSC‐CM short‐term proliferation. A) Schematic of EV bioactivity assays on hiPSC‐CM. hiPSC‐CM were plated at a third of the normal seeding density (≈100 000 cell cm^−2^) and treated with EV 24 h post‐seeding (0 h). Proliferation was assessed 24 and 48 h after treatment. B,C) Expansion of hiPSC‐CM represented as percentage increase over the untreated control (culture medium without added EV) at 24 and 48 h after treatment, respectively. Eight to nine fractions of a blank gradient were used as vehicle control. D) Immunofluorescence images and E) the quantification of proliferation marker EdU (red), cardiac troponin T (cTnT) (green), and nuclei (blue) in cardiomyocytes. F) Immunofluorescence images and (G) the quantification of mitotic cardiomyocytes assessed by phospho‐Histone H3 (phH3) (red), cTnT (green), and nuclei (blue). Data are mean ± SD [*n* = 3 in (B) and (C) and *n* = 4 in (E) and (G)]. Significance was tested against the negative control. *p < 0.05, ****p < 0.0001, n.s. (*p* > 0.05) by one‐way ANOVA with Dunnett's multiple comparisons test, with a single pooled variance.

We then investigated whether EV could exert a pro‐proliferative effect on hiPSC‐CM (**Figure** **4**A). When treated with hiPSC‐ and CPC‐EV, an increase in hiPSC‐CM number was observed compared to medium alone (untreated control) (Figure 4B), effect that was less pronounced at 48 h (Figure 4C). Interestingly, the increase in cardiomyocyte number was only supported by the concomitant increase in cell cycle activity, measured by cell cycle markers EdU and phospho Histone H3 (phH3), in hiPSC‐EV treated cells (Figure 4D–G). These results indicate that hiPSC‐EV may promote cardiomyocyte cell cycle‐reentry.

Notably, FBS‐derived EV, included as control throughout the bioactivity tests as a non‐relevant EV population for cardiac regenerative purposes, were unable to generate an angiogenic or a pro‐proliferative response in any of the assays, suggesting EV activity is not ubiquitous. Moreover, the vehicle control, used to monitor the background signal of the EV carrier solution, showed no effect on all bioactivity assays, confirming its inertness.

### 2.4 EV‐miRNA Reflects the Molecular Characteristics of the EV's Secreting Cell

**Figure 5:**
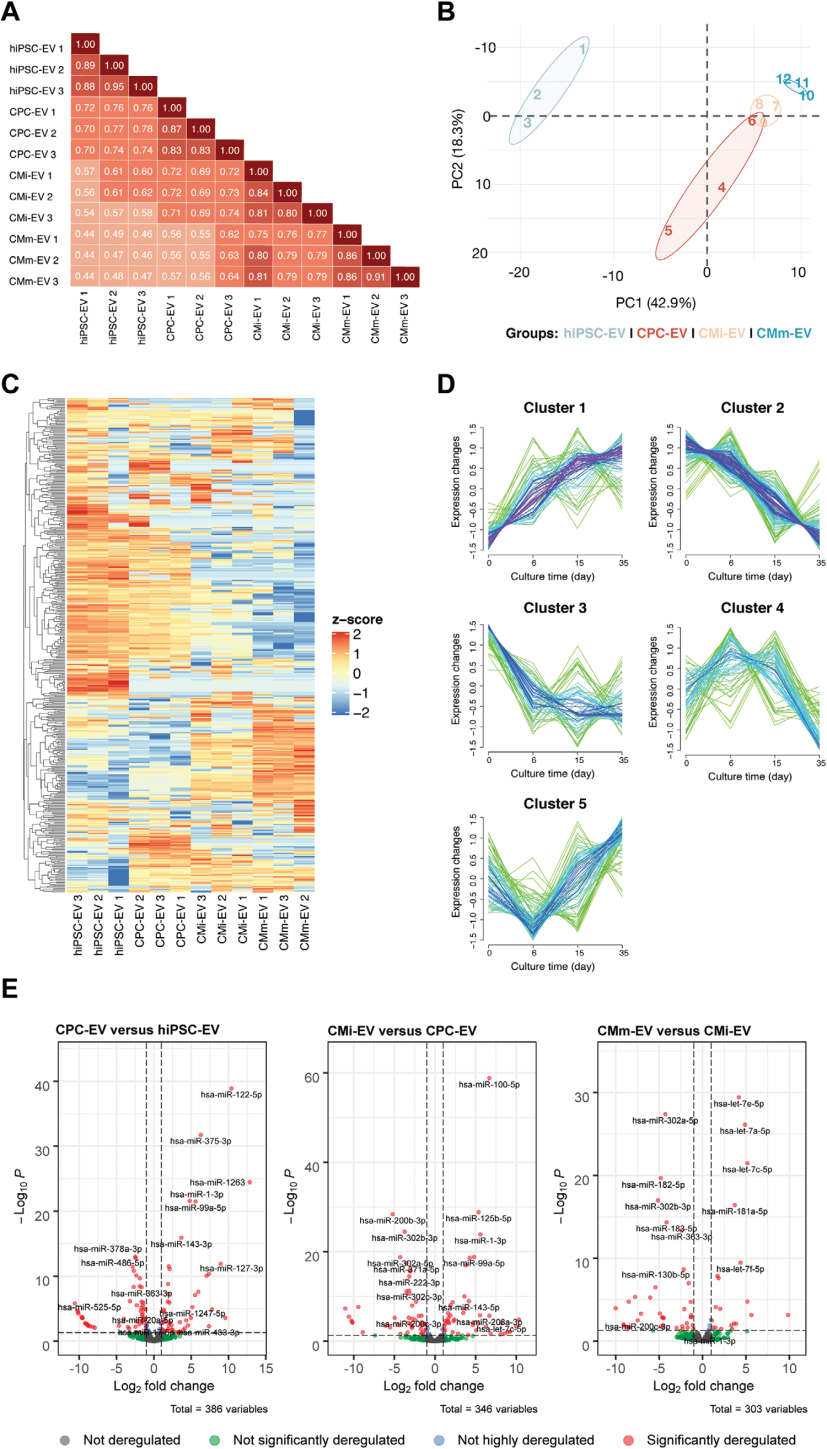
EV miRNome reflects the cellular changes that occur during hiPSC‐CM differentiation. A) Pearson's correlation analysis revealed a moderate degree of similarity between the miRNA expression profiles of all EV populations. B) Principal component analysis (PCA) shows a highly significant discrimination of the miRNA expressed between EV from different cell populations. C) Heatmap representing the *z*‐score normalized global miRNA expression in three biological replicates of hiPSC‐EV, CPC‐EV, CMi‐EV, and CMm‐EV, with a number of reads greater than a mean of 5 in at least one EV population. Dendrograms are based on complete‐linkage hierarchical clustering and Euclidean distances. D) Fuzzy plots representing the dynamic expression of five distinguishable miRNA clusters found in EV along hiPSC‐CM. E) Volcano Plots with pairwise comparisons of significantly differentially expressed miRNAs between EV populations (Log FC ≥ |−1| and *p* ≤ 0.05). All results correspond to EV from three independent isolations, corresponding to three different batches of conditioned culture media (*n* = 3).

To investigate the spectrum of the EV‐miRNA cargo and reveal variations in content underlying disparities in bioactivity, we performed next‐generation miRNA sequencing of EV. Comparing the miRNA content among EV groups, strong positive correlations were found between biological replicates within samples, suggesting consistency in EV production and isolation runs, and moderate positive correlations between different groups, with the greatest dissimilarity occurring between CMm‐EV and both hiPSC‐EV and CPC‐EV (**Figure** **5**A). Dimension reduction using principal component analysis (PCA) was used to explore broad miRNA differences between the four EV sets (Figure 5B). Results cluster each independent replicate together for each EV group, though with a higher dispersion for CPC‐EV. Principal component 1 (PC1), explaining 42.9% of the variance, separates EV secreted by hiPSC from those derived from cardiac committed populations. Principal component 2 (PC2), representing 18.3% of the variance, mainly separates CPC‐EV from the remaining EV groups.

We found 449 miRNAs expressed in at least one of these groups (based on the expression of at least five counts per million in each replicate). Global miRNA expression differed significantly across EV from different cell populations. Even considering intragroup variations, a consistent pattern can be observed throughout differentiation (Figure 5C). Fuzzy clustering^[^ ^49^ ^]^ was used to analyze these dynamic expression variations and led to the identification of five distinct miRNA clusters (Figure 5D, File S1, Supporting Information). One miRNA cluster with low expression in hiPSC‐EV increases upon differentiation and continues to increase throughout cardiomyocyte maturation. Another follows the opposite behavior, rapidly declining upon differentiation and continuing to fall until CMm‐EV. A third cluster declines after differentiation and remains stable throughout the remaining time‐points. A fourth cluster increases in CPC‐EV and CMi‐EV, and abruptly declines in CMm‐EV, while a fifth cluster decreases in CPC‐EV but rises in cardiomyocytes. Cluster optimization is shown in Figure S5A,B (Supporting Information). Similar expression profiles had previously been identified for hiPSC, primitive differentiated mesoderm progenitor cells, and cardiomyocytes.^[^ ^50^ ^]^

EdgeR^[^ ^51^ ^]^ was used to identify differentially expressed (DE) miRNA in EV secreted along hiPSC‐CM differentiation (counts per million at a minimum of five row counts, log fold change of ≥1 or ≤−1 at *p*‐value and FDR of ≤0.05). To achieve this, each differentiation stage was compared to its predecessor (CPC‐EV vs hiPSC‐EV; CMi‐EV vs CPC‐EV; CMm‐EV vs CMi‐EV). Overall, the highest number of DE miRNA was observed in EV corresponding to the cellular transition from pluripotent to cardiac committed cells (CPC‐EV vs hiPSC‐EV), while CMi and CMm‐EV presented the smallest number of DE miRNA, indicating a stabilization of the EV miRNome. In particular, we identified 55 downregulated and 35 upregulated miRNAs in CPC‐EV versus hiPSC‐EV (Table S1, Supporting Information); 41 downregulated and 39 upregulated miRNAs in CMi‐EV versus CPC‐EV (Table S2, Supporting Information); and 23 downregulated and 13 upregulated miRNAs in CMm versus CMi‐EV (Table S3, Supporting Information).

Volcano plots were generated by pairwise comparison of the four cell populations (Figure 5E). Expression patterns of pluripotency‐associated and cardiac development‐associated miRNAs are shown on Figures S6A,B (Supporting Information), correspondingly.

### 2.5 A miRNA Subset Is Differentially Expressed in EV throughout hiPSC‐CM Differentiation

**Figure 6:**
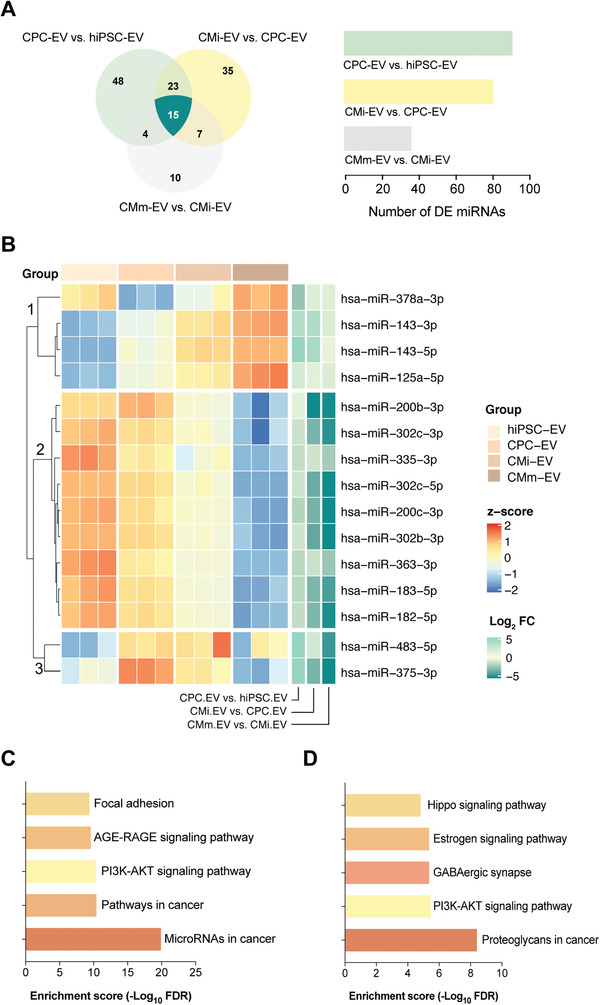
An EV‐miRNA cluster is differentially expressed throughout hiPSC‐CM differentiation. A) Venn diagram representation of the unique and shared differentially regulated miRNA between CPC‐EV versus hiPSC‐EV (light green), CMi‐EV versus CPC‐EV (yellow), and CMm‐EV versus CMi‐EV (gray). B) Heatmap and dendrograms of *z*‐score normalized miRNA expression levels illustrating the 15 common differentially expressed miRNA obtained in (A). miRNAs are clustered based on k‐means (clusters 1, 2, and 3). Fold changes (in log scale) are shown on the right side of the plot. C,D) Pathway enrichment analysis for miRNA in cluster 2 of the heatmap shown in (B). C) Top 5 KEGG (Kyoto Encyclopedia of Genes and Genomes) pathways for experimentally validated targets of miRNA obtained with Ingenuity Pathway Analysis. D) Top 5 KEGG pathways for miRNA obtained with DIANA‐miRPath v3.0.

Once we characterized the landscape of EV‐miRNA expression, we investigated the 15 miRNA that were DE throughout cardiomyocyte differentiation and maturation (**Figure** **6**A). K‐means clustering revealed distinguishable miRNA expression patterns among the samples, with three main miRNA subsets emerging (Figure 6B). miRNA from the first cluster (miR‐378a, miR‐143, miR 125a), strongly expressed in the mammalian heart, and largely involved in muscle development and hypertrophy,^[^ ^52^, ^53^, ^54^, ^55^, ^56^ ^]^ were downregulated in hiPSC‐EV and gradually upregulated along cardiomyocyte differentiation (with exception of miR‐378a, which is also expressed in hiPSC‐EV). The second miRNA cluster (miR‐200b/c, miR‐335, miR‐302b/c, miR‐363, miR‐183, miR‐182), contained pluripotency‐associated miRNAs,^[^ ^31^, ^32^, ^33^, ^57^ ^]^ which were progressively downregulated in EV secreted from committed and mature cells. The third cluster is composed solely of two miRNAs (miR‐483, miR‐375), involved in lineage specification, which explains their absence in hiPSC‐EV and CMm‐EV.^[^ ^58^ ^]^ miR‐483 is induced during mesoderm formation, and is in accordance with a differentiation toward a cardiac lineage.^[^ ^58^ ^]^ However, miR‐375 is implicated in definitive endoderm specification, and its presence in CPC‐ and CMi‐EV is unexpected, although previous studies had already identified it in hiPSC‐CM.^[^ ^14^ ^]^

Given the in vitro bioactivity results, to predict cellular pathways affected by hiPSC‐EV, we used both Ingenuity Pathway Analysis (IPA) and DIANA‐miRPath v3.0^[^ ^59^ ^]^ to map the targets of the second miRNA subset which are particularly enriched in hiPSC‐EV. Using IPA, seven out of the nine input microRNAs had targeting information available, of which six had experimentally validated data for 164 mRNA targets. We tested these target genes for overrepresentation in biological pathways, defined by KEGG terms (Figure 6C). To corroborate these results, the same nine miRNAs with identified targets in IPA were run through DIANA‐miRPath v3.0,^[^ ^59^ ^]^ which performs direct functional annotation on input miRNA (Figure 6D). The PI3K/AKT pathway was present in the top five overrepresented KEGG pathways identified using both target prediction methods (Figure 6C,D), and directly targeted by five miRNAs (Table S4, Supporting Information).

### 2.6 hiPSC‐EV Mediate AKT Activation in Recipient Endothelial Cells

**Figure 7:**
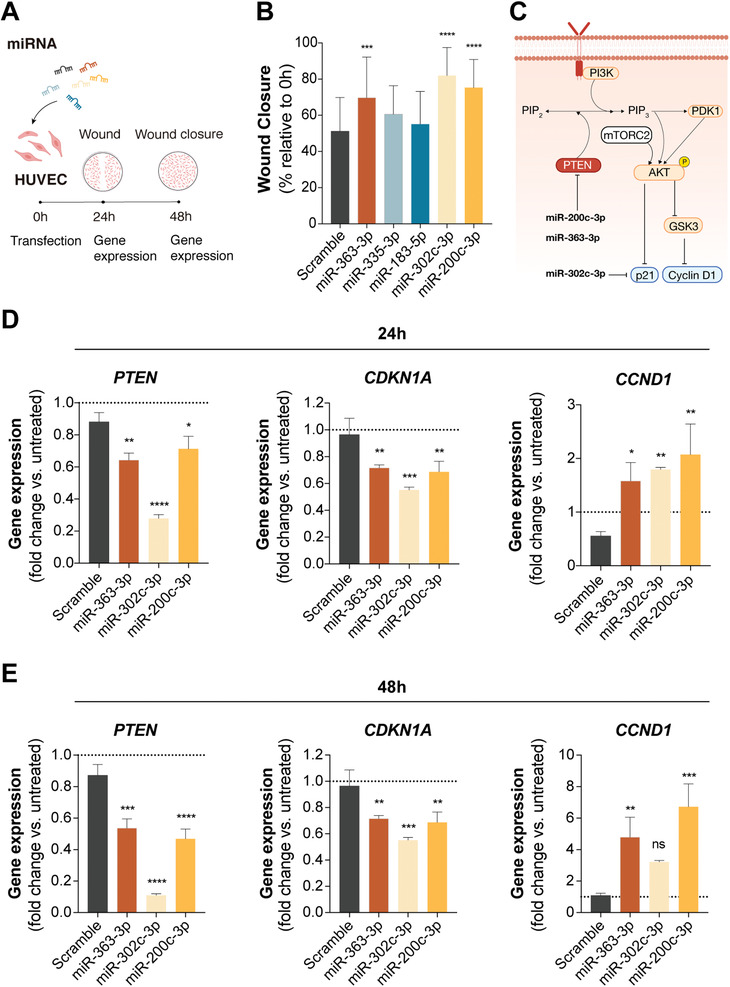
Overexpression of a miRNA stemness cluster induces endothelial cell migration. A) Schematic representation of the transient transfection assays performed on human umbilical vein endothelial cells (HUVEC). B) Wound closure 24 h post‐scratch (48 h post‐transfection). Three of the five miRNA transfected into HUVEC were able to promote wound closure (measured as a percentage of the initial wound area; *n* = 4). C) Schematic overview of the described interactions of miRs‐200c‐3p, 363‐3p, and 302c‐3p with the PI3K/AKT pathway (Adobe Illustrator). D,E) Gene expression profiles of *PTEN*, *CDKN1A* (p21^WAF/Cip1^), and *CCND1* (cyclin D1) at 24 and 48 h after HUVEC transient transfection. Data presented as mean ± SD, *n* = 4 for (B) and *n* = 3 for (D) and (E). Significance was tested against the scramble control. *p < 0.05, **p < 0.01, ***p < 0.001, ****p < 0.0001, n.s. (*p* > 0.05) by one‐way ANOVA with Dunnett's multiple comparisons test, with a single pooled variance.

To further explore the effect of hiPSC‐EV on cardiac regeneration, overexpression of the 5 DE miRNAs identified by IPA to directly target the PI3K/AKT pathway was evaluated on HUVEC (**Figure** **7**A). Transient miRNA overexpression was achieved by lipid‐base transfection of miRNA mimics (sequences available in Table S7, Supporting Information). Transfection efficiency was estimated to be ≈60%, by RT‐qPCR detection of *TWF1*, targeted by the positive transfection control miR‐1 (data not shown). We observed that upon transient transfection only three out of the five miRNAs were able to exert a significant effect on endothelial cell migration (Figure 7B). miR‐200c‐3p and miR‐363‐3p directly target *PTEN* mRNA, a major homeostatic regulator and tumor suppressor protein, while miR‐302c‐3p targets the cyclin‐dependent kinase inhibitor *CDKN1A* (p21^WAF/Cip1^) (Figure 7C). RT‐qPCR analysis of HUVEC transfected with these miRNAs confirmed downregulation of the corresponding mRNA targets at 24 and 48 h post‐transfection (Figure 7D,E, respectively). Remarkably, miR‐302c‐3p also caused a significant downregulation of *PTEN*, although this direct interaction was not described in IPA. *PTEN* downregulation was accompanied with suppression of *CDKN1A* (p21^WAF/Cip1^) and upregulation of *CCND1* (cyclin D1) (Figure 7D,E), both downstream effectors of AKT (Figure 7C), and important regulators of cell proliferation, with opposing roles on cell cycle progression.^[^ ^60^ ^]^

**Figure 8:**
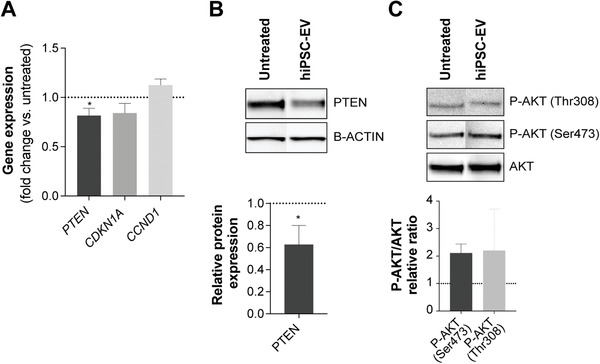
hiPSC‐EV target the PI3K/AKT pathway. A) Gene expression of *PTEN*, *CDKN1A* (p21^WAF/Cip1^), and *CCND1* (cyclin D1) on HUVEC treated with hiPSC‐EV for 24 h. B) Western blot for PTEN and quantification of PTEN expression relative to B‐ACTIN. C) Western blot for total AKT and active phosphorylated forms (P‐AKT Thr308 and P‐AKT Ser473). Relative quantification of P‐AKT was performed against total AKT. Data are mean ± SD, *n* = 3. Relative ratios were calculated in relation to the untreated control (ECGM‐2 only). Significance tested by a one‐sample *t*‐test. *p < 0.05.

We then hypothesized that hiPSC‐EV mediated bioactivity would, at least partially, rely on activation of the PI3K/AKT pathway. In fact, HUVEC treated with hiPSC‐EV showed a significant reduction in *PTEN* gene expression (18.4 ± 7.3%) after 24 h (**Figure** **8**A) when compared to the untreated group, effect that was further confirmed at the protein level (37.3 ± 17.3%) (Figure 8B). A tendency to downregulate *CDKN1A* and upregulate *CCND1* expression could also be observed, although these results were not statistically significant (Figure 8A). Still, an increase in AKT activity, measured by its level of phosphorylation at both Thr308 and Ser473 sites, was detected (Figure 8C and Figure S7, Supporting Information).

## 3 Discussion

EV play a major role in cardiac repair processes,^[^ ^5^, ^9^, ^61^ ^]^ making them an attractive therapy to boost cardiac regeneration. In this context, identification of the EV(s) and corresponding cell platform(s) more suitable for therapeutic application, as well as characterization of these EVs’ genetic signatures is paramount. In this work, we studied the potential of EV secreted along hiPSC‐CM differentiation to induce angiogenesis and cardiomyocyte proliferation, two key mechanisms required for recovery of cardiac function post‐acute myocardial infarction. Moreover, we comprehensively investigated the EV miRNome along these stages, and identified DE miRNAs that may underlie EV bioactivity. Through a combination of functional assays in endothelial cells and cardiomyocytes, we showed that hiPSC‐EV provide a higher therapeutic benefit in vitro, in comparison to EV from hiPSC cardiac‐committed derivatives, and that this effect is possibly attained through the activity of pluripotency‐associated miRNAs.

Previous studies have reported the cardiac regenerative effects of EV derived from various cell sources.^[^ ^14^, ^62^, ^63^, ^64^, ^65^ ^]^ Most of these studies have focused on CPC‐ or cardiomyocyte‐derived EV, based on the premise that the cargo of partially, or terminally, differentiated cells would be enriched in miRNAs that modulate cardiac‐specific processes, as opposed to miRNA from undifferentiated cells.^[^ ^14^ ^]^ In fact, reports on the functionality of EV secreted from hiPSC present conflicting data, either finding no relevant activity,^[^ ^14^ ^]^ or showing important cardioprotective properties for these EV.^[^ ^9^, ^66^, ^67^ ^]^ A similar trend can be observed for cardiomyocyte‐EV, with studies reporting their negligible secretion and consequent therapeutic irrelevancy,^[^ ^10^ ^]^ while others describe them as significant anti‐apoptotic,^[^ ^14^ ^]^ angiogenic,^[^ ^29^ ^]^ or autophagy^[^ ^30^ ^]^ modulating agents. Most of these differences may be explained by disparities in cell source, and variations in EV separation, purification and characterization methods between studies, that may lead to the isolation of different EV subtypes, ultimately impacting downstream analysis.^[^ ^68^, ^69^ ^]^ By providing a detailed comparison between hiPSC‐, CPC‐, CMi‐, and CMm‐EV derived from a continuous differentiation process from the same cell source, isolated and characterized through a consistent workflow, we eliminate some of these biases.

We corroborated a pattern in EV‐miRNA content indicative of stem cell to cardiomyocyte specification, confirming that EV cargo reflects the molecular characteristics and cellular stage of its parent cell. Several of the miRNA enriched in hiPSC‐EV belong to stemness maintenance miRNA clusters, whose expression specifically characterizes human pluripotent stem cells, such as the miR‐302a‐367 cluster^[^ ^33^ ^]^ (miR‐302c, miR‐302b), paralogue clusters miR‐17‐92 cluster^[^ ^31^, ^57^ ^]^ (miR‐17, miR‐20a) and miR‐106a–363^[^ ^31^ ^]^ (miR‐106a, miR‐20b, miR‐363), the chromosome 19 miRNA cluster (C19MC)^[^ ^34^ ^]^ (miR‐512, miR‐525, miR‐520a, miR‐1323), and miR‐200c,^[^ ^33^ ^]^ which does not belong to any of the aforementioned families. As expected, expression of these miRNA consistently declines in EV, in response to early differentiation cues, and over the differentiation time course, as had previously been observed for hiPSC.^[^ ^70^ ^]^ CPC‐EV show a downregulation of pluripotency‐associated miRNAs, and an increase expression in miRNA induced upon commitment, such as miR‐483, miR‐654, miR‐1247, miR‐146b, miR‐3605, involved in signaling of cardiac mesoderm formation,^[^ ^50^, ^58^ ^]^ as well as myomiRs important for cardiac specification (as miR‐1, miR‐133a and miR‐143).^[^ ^50^ ^]^ Analysis of EV secreted from cardiomyocytes cultured up to day 35 (CMm) indicates that cells continue to mature toward a more adult‐like gene expression pattern, with increasing expression of cardiomyocyte‐specific myomiRs (miR‐1, miR‐133a, miR‐143, miR‐145, miR‐208a/b),^[^ ^50^, ^71^ ^]^ the let‐7 family members,^[^ ^72^ ^]^ and miR‐378a,^[^ ^73^ ^]^ important players in late cardiogenic stages and cardiomyocyte maturation. The identification of the miRNAs involved in the hiPSC to hiPSC‐CM cellular transitions in their secreted EV adds a new layer of complexity to our understanding of hiPSC differentiation and cardiac development and may be used as a nondestructive method for characterization and monitoring of cells’ identity and potency.

Remarkably, a particular miRNA subset was found to be DE along hiPSC‐CM differentiation and maturation. Even though our primary goal was the characterization of the EV‐miRNA landscape across hiPSC‐CM differentiation, and not the selection of unique individual miRNAs, we chose three miRNAs belonging to this cluster (miR‐200c‐3p, miR‐363‐3p, and miR‐302c‐3p), highly expressed in hiPSC‐EV and consistently downregulated in cardiac cell derivatives, to study in further detail. Overexpression of these miRNAs was found to promote endothelial cell migration by activation of the PI3K/AKT pathway. Other studies had already reported the role of these miRNA in the cardiac context. Jung et al. reported an increase in miR‐106a‐363 cluster in EV secreted by hypoxic iPSC‐CM.^[^ ^74^ ^]^ The authors showed cardiomyocyte‐EV enriched in this cluster stimulated cell cycle re‐entry of cardiomyocytes by repressing the Notch3 signaling pathway.^[^ ^74^ ^]^ Tian et al. demonstrated that the miR‐302‐367 cluster is important for cardiomyocyte proliferation during development, and sufficient to induce cardiomyocyte proliferation and promote regeneration in the adult heart. In a mice model, the authors showed that increased miR‐302‐367 expression resulted in a significant cardiomyocyte proliferation, possibly through repression of the Hippo signal transduction pathway.^[^ ^75^ ^]^ Both these clusters were identified by Diez‐Cuñado et al. as able to promote cardiomyocyte proliferation.^[^ ^76^ ^]^ The role of miR‐200c is less clear, being mostly associated with cardiomyocyte hypertrophy.^[^ ^52^ ^]^ Overall, we can conclude that most of the miRNAs associated with cell cycle progression, regulation of apoptosis and angiogenesis, converge on hiPSC‐EV^[^ ^77^, ^78^ ^]^ corroborating its pro‐angiogenic and pro‐proliferative properties.

We propose that, mechanistically, the PI3K/AKT pathway may be part of the cellular signaling cascade involved in native hiPSC‐EV bioactivity, as several stemness maintenance miRNA enriched in hiPSC‐EV were predicted to target diverse molecules along this pathway. As previously reviewed,^[^ ^79^ ^]^ the PI3K/AKT pathway plays a crucial role in cell metabolism, proliferation, survival, and migration. Moreover, we observed that PTEN, a major homeostatic and tumor suppressor protein, was downregulated in endothelial cells treated with hiPSC‐EV. This hiPSC‐EV mediated suppression of PTEN led to an increase in phosphorylation of AKT, thereby promoting angiogenesis and proliferation via inactivation of CDK inhibitor p21^WAF/Cip1^ and indirect activation of cyclin D1, both important regulators in the G1 to S phase cell cycle progression in several cell types.^[^ ^60^ ^]^ While the magnitude of hiPSC‐EV effect on p21^WAF/Cip1^ and cyclin D1 was not large, it may be sufficient to explain the mild cell proliferation results obtained with a single short‐term treatment in cardiac cells. Of note, cardiomyocyte proliferation assays were performed with hiPSC‐CM and not adult cardiomyocytes. Even though in vitro proliferation declines with maturation, hiPSC‐CM still show immature features and a small degree of proliferation and thus it may be easier to induce cell‐cycle reentry in these cells, compared to adult ones.

Dysregulation of the PTEN/PI3K/AKT signaling pathway is implicated in a number of diseases including cancer, where *PTEN* mutations and deletions consequently lead to increased cell proliferation and reduced cell death.^[^ ^80^ ^]^ Our findings complement recent studies in which PTEN partial inhibition, achieved either through small molecules, gene knockdown, or miRNAs led to increased cardiomyocyte proliferation^[^ ^81^ ^]^ and reduced apoptosis^[^ ^82^ ^]^ in vitro, and attenuation of endothelial cell apoptosis^[^ ^83^ ^]^ and overall improved cardiac outcomes^[^ ^84^ ^]^ in vivo. The PI3K/AKT pathway also mediates other pro‐survival mechanisms unexplored in this work, namely inhibition of pro‐apoptotic proteins, such as BAD,^[^ ^85^ ^]^ and inhibition of pro‐apoptotic signals generated by transcription factors like FOXO1.^[^ ^85^ ^]^

In vitro cell‐based assays as the ones employed in this study do not accurately replicate the physiological or pathological conditions of the human heart but are useful predictive tools for screening of therapeutic molecules.^[^ ^86^ ^]^ Nonetheless, further studies, including in vivo experiments, are needed to understand the mechanism of action and the exact role of hiPSC‐EV in cardiac regeneration. In fact, the properties and cargo of hiPSC‐EV may vary according to the iPSC line used, and thus may not be generalized to all hiPSC. Here, we compared two hiPSC lines regarding bioactivity in endothelial cells and showed that, although the same tendency can be observed for both, results were more significant for hiPSC1. Therefore, the possibility that hiPSC‐, CPC‐, CMi‐ and CMm‐EV will show tropism toward specific cell types in vivo and exert different effects than the ones showed here should not be excluded.

Finally, the impact of EV on inflammation and fibrosis should also be addressed. Cardiac fibroblasts are major players in the formation of the fibrotic, non‐functional scar tissue after acute myocardial infarction, process also modulated by cardiac‐resident macrophages.^[^ ^41^ ^]^ In vitro and preclinical studies showed cardiosphere‐derived EV were able to attenuate cardiac fibrosis^[^ ^87^ ^]^ and modulate immune responses, such as inducing macrophage polarization^[^ ^88^ ^]^ or regulating cytokine secretion.^[^ ^89^ ^]^ However, evidence for the role of hiPSC‐EV in these processes is still lacking^[^ ^9^, ^90^ ^]^ and should be addressed in future in vivo studies, as reliable and predictive in vitro models of cardiac fibrosis able to respond to fibrotic and anti‐fibrotic stimulation are yet to be standardized and currently do not reflect the interaction between fibroblasts, macrophages and endothelial cells, major players driving myocardial fibrosis.^[^ ^91^, ^92^ ^]^

## 4 Conclusions

This study provides a comprehensive characterization of EV secreted throughout hiPSC differentiation and maturation toward cardiomyocytes. We identified the miRNA profiles involved in hiPSC‐CM cellular transitions to cardiomyocyte commitment, in their secreted EV. We demonstrated that hiPSC secrete bioactive EV, enriched in pluripotency‐associated miRNA, with higher in vitro pro‐angiogenic and pro‐proliferative properties, in comparison to their partially and terminally committed counterparts. Moreover, our data suggest that inhibition of PTEN and activation of the PI3K/AKT pathway may be part of the cellular signaling cascade involved in hiPSC‐EV‐induced migration in endothelial cells. Although further work is required to completely elucidate the mechanisms of hiPSC‐EV function, our study proposes hiPSC as relevant candidates for the production of EV‐based therapies for cardiac regeneration.

## 5 Experimental Section

### hiPSC Culture

Two hiPSC lines, namely, IMR90‐4 (hereafter referred as hiPSC1) and DF19‐9‐11T.H (hereafter referred as hiPSC2) (both from WiCell) were used for this study. Cells were expanded on coated plates (Matrigel hESC‐Qualified Matrix, Corning), in feeder‐free and animal component‐free TeSR‐E8 medium (Stemcell Technologies), in a humidified atmosphere at 37 °C and 5% CO_2_. Both hiPSC lines tested negative for mycoplasma contamination. Results from hiPSC2 are shown in Supporting Information, except where stated.

### hiPSC Differentiation toward Cardiomyocytes

hiPSC differentiation was initiated when cells reached 80%–90% confluency, by temporal modulation of the Wnt/ß‐catenin signaling following a protocol previously described by the authors.^[^ ^93^, ^94^ ^]^ Briefly, expansion medium (TeSR‐E8) was replaced by differentiation media (RPMI 1640 (Gibco, ThermoFisher Scientific) with B27 without insulin (ThermoFisher Scientific) (RPMI + B27‐I), supplemented with CHIR99021 (12 × 10^−6^ m) (Tocris Bioscience), Activin A (80 ng mL^–1^) (Tebu‐bio), and ascorbic acid (50 µg mL^–1^) (Sigma Aldrich). After 24 h, the medium was replaced by differentiation medium supplemented with IWR1 (5 × 10^−6^ m) (Selleckchem) and ascorbic acid (50 µg mL^–1^). At day 3 (72 h after differentiation induction), medium was exchanged for differentiation medium supplemented with IWR‐1 (5 × 10^−6^ m). At day 6, medium was replaced by maintenance medium [RPMI 1640 supplemented with B27 with insulin (RPMI + B27)].

### Generation of hiPSC‐CM Aggregates

Between days 7 and 8 (when over 80% of the cells were beating), cells were dissociated by incubation with TrypLE Select (Gibco, ThermoFisher Scientific) for 8 min and aggregated using AggreWell 400 plates (Stemcell Technologies), according to the protocol described by Correia et al.^[^ ^44^ ^]^ Forty‐eight hours after aggregation, CM aggregates were harvested and transferred to an orbital suspension culture system, at an agitation rate of 90 rpm. Maintenance medium was changed every other day thereafter.

### hiPSC‐CM Maturation

At day 15 of culture RPMI + B27 medium was replaced by maturation medium, composed of RPMI 1640 without glucose (MP biomedicals, ThermoFisher Scientific) supplemented with B27, 1 × 10^−3^ m of glutamine (Sigma‐Aldrich), 10 × 10^−3^ m of d(+)‐galactose (Sigma‐Aldrich), 100 × 10^−6^ m of oleic acid (Sigma‐Aldrich), and 50 × 10^−6^ m of palmitic acid (Sigma‐Aldrich), to promote cardiomyocyte metabolic maturation. Maturation medium was changed every other day thereafter. Cardiomyocyte aggregates were cultured in these conditions for an additional 20 d, reaching a total of 35 d of culture, since the start of the differentiation.^[^ ^45^ ^]^

### Medium Harvesting

An equal volume (240 mL) of conditioned culture medium (CondM) was harvested at days 0 (expansion medium), 6 (differentiation medium), 15 (maintenance medium), and 35 (maturation medium) of culture, corresponding to 24, 72, 48, and 48 h of EV production correspondingly. At each timepoint of medium harvest, total cell number and viability were estimated by trypan blue exclusion assay. Cell viability was also assessed through cell membrane integrity analysis by incubation of cell samples with enzyme substrate fluorescein diacetate (FDA, Sigma‐Aldrich) and DNA‐binding dye propidium iodide (PI, Sigma‐Aldrich). Phase contrast and fluorescence images were acquired using an inverted fluorescence microscope (DMI6000, Leica Microsystems GmbH) and analyzed with ImageJ open‐source software.^[^ ^95^ ^]^

### EV Separation from Conditioned Culture Medium

EV were separated from CondM by differential centrifugation followed by density gradient ultracentrifugation. Briefly, immediately after harvest, CondM was centrifuged twice at low speeds (10 min at 300*g*, followed by a second centrifugation for 10 min at 2000*g*; rotor A‐4‐81, 5810 R centrifuge, Eppendorf) to remove major contaminants. The resulting supernatant was filtered through 0.45 × 10^−6^ m filter units (Nalgene Rapid‐Flow, Thermo Fisher Scientific) and ultracentrifuged in 30 mL conical open‐top polyallomer tubes (Beckman Coulter) for 3 h using a XL‐100 ultracentrifuge (SW 28 rotor, Beckman‐Coulter) at 110 000*g_max_* to create an EV pellet, hereby called 100K pellet. An OptiPrep density gradient (ODG, Axis Xield Diagnostics) was prepared as reported by Van Deun et al. with minor modifications.^[^ ^68^ ^]^ An iodixanol working solution (50% iodixanol) was made by adding a working solution buffer (60 × 10^−3^ m Tris‐HCl, 6 × 10^−3^ m EDTA, 0.25 m sucrose, pH 7.4) to a stock solution of OptiPrep (60% w/v aqueous iodixanol solution). Appropriate amounts of a homogenization buffer [10 × 10^−3^ m tromethamine–hydrochloric acid (Tris‐HCl), 1 × 10^−3^ m ethylenediaminetetraacetic acid (EDTA), and 0.25 m sucrose, pH 7.4] and the iodixanol working solution were mixed to prepare 5%, 10%, and 20% iodixanol solutions. The 100K pellet was mixed with the 50% iodixanol working solution, to create a 40% EV‐containing solution. The gradient was prepared by consecutive layering 4 mL of 40% (containing the EV sample), 4 mL of 20%, 4 mL of 10%, 3.5 mL of 5% iodixanol solutions, and 1 mL of PBS from the bottom to the top of a 16.8 mL open‐top polyallomer tube (Beckman Coulter), creating a bottom‐up ODG, where EV migrated upward. The gradient was centrifuged at 4 °C for 18 h at 110 000*g_max_* (SW 28.1 rotor, Beckman Coulter). After centrifugation, 16 fractions of 1 mL were collected from top (fraction 1) to bottom (fraction 16), and divided into six samples by pooling fractions 1–4, 5–7, 8–9, 10–12, and 13–16. Pooled gradient fractions were concentrated to 300 µL using Amicon Ultra‐2 mL 10 kDa filter units (Merck Millipore). The resulting samples were aliquoted and stored at ‐80 °C until further characterization. EV were mostly found in fractions 8 and 9, as previously reported.^[^ ^68^, ^96^ ^]^ This isolation procedure was repeated with DPBS (Gibco, Thermo Fisher Scientific) as negative control and Fetal Bovine Serum (FBS qualified, EU‐approved, Gibco, Thermo Fisher Scientific) as positive control, as well as with the medium used for cell culturing at each stage, to provide a characterization of the background particles already present in the non‐CondM.

### EV Density Determination

A digital refractometer AR200 (Leica Microsystems GmbH, Wetzlar, Germany) was used to measure the refractive index of EV‐rich fractions (8–9). The refractive index was converted to density based on a standard curve ranging from 10% to 30% iodixanol.

### Transmission Electron Microscopy and Immunogold Labeling

EV‐rich fractions (8–9) were subjected to transmission electron microscopy to assess the presence and morphology of EV. A drop (3 µL) of sample was adhered to pre‐coated formvar/carbon/glow discharged 100 mesh copper‐palladium grids for 20 min. Grids were fixed with 2% formaldehyde (Science Services) in 0.1 m phosphate buffer for 20 min, washed with sterile water and stained with 2% uranyl acetate for 5 min. For immunogold labeling, after fixation, the grids were immersed in 50 × 10^−3^ m glycine in PBS for 15 min and blocked for 10 min with 10% fetal calf serum (FCS) in PBS. Without rinsing, the grids were immediately placed into the primary antibody (1:200 diluted in 5% FCS in PBS, anti‐CD63 ab59479, Abcam) for 30 min at RT. As control, some of the grids were not exposed to the primary antibody. Grids were rinsed with 0.5% FCS in PBS and incubated with 12 nm colloidal gold‐AffiniPure goat‐anti‐mouse IgG secondary antibody (1:20 diluted in 5% FCS in PBS, 115‐205‐146, Jackson Immunoresearch) for 30 min at RT. The grids were rinsed with PBS and post‐fixed in 1% glutaraldehyde (Science Services) for 5 min. After rinsing in distilled water, the grids were stained using 2% uranyl acetate for 5 min. Grids were examined using electron microscopy (FEI Tecnai G2 Spirit BioTWIN operating at 120 keV equipped with an Olympus‐SIS Veleta CCD Camera).

### Nanoparticle Tracking Analysis

NTA was performed using a NanoSight NS300 (Malvern Instruments Ltd.), equipped with a 488 nm laser (<55 mW maximum power) and an automatic syringe pump system. NTA was used to measure the size, size distribution and concentration of the particles from pooled Optiprep density gradient EV rich fractions (8–9). For each individual sample, three videos of 60 s were recorded with screen gain 2 and camera level 14 and a syringe pump infusion speed of 40. All videos were analyzed by NTA software version 3.3 with screen gain 10 and detection threshold 3. To achieve optimal measurements, samples were diluted with DPBS to obtain a particle concentration within the optimal range of the NTA software (3 × 10^8^–1 × 10^9^ particle mL^−1^). All size distributions determined with NTA correspond to the hydrodynamic diameters of the particles in suspension.

### Western Blotting

To assess the presence of EV in all pooled gradient fractions (1–4, 5–7, 8–9, 10–12, 13–16) harvested after ODG ultracentrifugation, western blot was performed for EV specific markers (CD63, CD81, and TSG101). Subsequently, the EV‐rich fractions (8–9) isolated from all cell types were subjected to western blot to identify EV by specific (CD63, TSG101, Flotilin‐2) and non‐specific (AGO2) EV markers. Whole cell lysates as well as 100K pellets were analyzed in parallel with EV samples, where stated. EV samples were normalized by volume (20 µL of sample per lane), while whole cell lysates were normalized by protein amount (10 µg of protein per lane). Protein concentrations of cell lysates were determined by microBCA (ThermoFisher Scientific). All samples were diluted in lysis buffer (NuPAGE LDS sample buffer 4×, Novex, Life Technologies Europe B.V.) with added reducing agent (for TSG101, Flotillin‐2, AGO2, and GM130) or in nonreducing conditions for tetraspanins (CD63 and CD81) and boiled for 5 min at 95 °C. Protein samples were separated by SDS polyacrylamide gel electrophoresis in MES running buffer (Novex, Life Technologies Europe B.V.) and subsequently transferred to a nitrocellulose membrane (iBlot Transfer Stack, nitrocellulose, mini, Novex, Life Technologies Europe B.V.). Immediately after transfer, membranes were stained with Ponceau S solution (Sigma Aldrich), and photographed. Membranes were de‐stained and blocked at RT with 5% skim milk in tris‐buffered saline + 0.1% tween 20 (TBST) for 1 h. Afterward, membranes were exposed to CD63 (1:1000, ab59479, Abcam, Cambridge, UK), CD81 (1:1000, EXOAb‐CD81A‐1, System Biosciences), TSG101 (1:1000, EXOAb TSG, System Biosciences), Flotillin‐2 (1:1000, 610383, BD), and AGO2 (1:1000, ab32381, Abcam) primary antibodies and incubated overnight at 4 °C. All antibody dilutions were performed on 5% skim milk prepared in TBST. Membranes were extensively washed with TBST and incubated with the appropriate secondary antibody (anti‐rabbit‐HRP, 1:20000, System Biosciences; anti‐mouse ECL, 1:5000, NA931, GE lifesciences). For signal detection, chemiluminescent substrate (WesternBright Sirius, Advansta) was added to the membranes according to the manufacturer's instructions. Imaging was performed using a ChemiDoc imaging system (Bio‐Rad Laboratories).

### EV Labeling with PKH26

EV labeling was performed as described by van der Vlist and colleagues, using the PKH26 Red Fluorescent Cell Linker Mini Kit for General Cell Membrane Labelling (Sigma Aldrich).^[^ ^97^ ^]^ Briefly, for every sample, 1.5 µL of PKH26 was diluted in 100 µL of diluent C. EV, resuspended in 20 µL of 0.2% bovine serum albumin (BSA) in DPBS, were diluted in 80 µL of diluent C, added to the diluted PKH26, and mixed by pipetting. The labeling reaction occurred for 3 min at room temperature and was stopped by the addition of 100 µL of 0.1% BSA.^[^ ^97^ ^]^ An equal protocol was followed for the preparation of a negative control, in which the EV sample was replaced by PBS. Stained vesicles (PKH26‐EV) and the corresponding control (PKH26‐PBS) were loaded on the bottom of a density gradient, and ultracentrifuged as previously described, to separate labeled particles from unbound dye.

### EV Uptake Assay

PKH26‐labeled EV were used to evaluate EV uptake into HUVEC and hiPSC‐CM. HUVEC and hiPSC‐CM were seeded at a density of 18 000 or 100 000 cell cm^−2^, respectively. After 24 h, cells were washed twice with DPBS and incubated with medium supplemented with PKH26‐EV, at a dose of 3000 particles per cell for HUVEC or 300 particles per cell for hiPSC‐CM, or with equal volume of control PKH26‐PBS, in the presence or absence of Dynasore (Sigma Aldrich), as previously reported by Chiba and colleagues.^[^ ^48^ ^]^ Following a 3 h incubation at 37 °C in 5% CO_2_, cells were washed twice with EV‐free medium to remove any extraneous labeled EV not internalized by cells, and subsequently fixed at room temperature for 20 min with 4% (w/v) paraformaldehyde. Nuclei were counterstained with 4′,6‐diamidino‐2‐phenylindole (DAPI), dihydrochloride, ThermoFisher Scientific) reagent in a 1:2000 dilution in DPBS. Fluorescence images were acquired using an inverted fluorescence microscope (DMI6000, Leica Microsystems GmbH). Quantification of signal intensity was performed using Fiji software.^[^ ^98^ ^]^ HUVEC were additionally stained for CD31 marker, to confirm their phenotype [anti‐CD31 antibody diluted in 0.2% (v/v) FSG, 1:50; clone JCF0A, M0823, DAKO Omnis, Agilent Technologies; mouse IgG isotype control, 1:50, sc‐3877, Santa Cruz Biotechnology].

### HUVEC Culture

Healthy HUVEC were purchased from Lonza (#C2517A, Lonza) and cultured according to the manufacturer's specifications. Briefly, cells were maintained in Endothelial Cell Growth Medium 2 (ECGM‐2, Promocell), containing 2% of FCS, in a humidified atmosphere at 37 °C in 5% CO_2_. Medium was changed every 48 h. Cells where subculture upon reaching 70%–85% confluency. HUVEC were only maintained and used up to passage 5, including.

### Tube Formation Assay

HUVEC were seeded at 12 000 cells per well onto 96‐well plates coated with 40 µL of basement membrane extract [Matrigel Growth Factor Reduced (GFR) Basement Membrane Matrix, Phenol Red‐Free, Corning, Corning Inc.]. 30 min after seeding, medium was replaced with EV‐supplemented medium, or the corresponding controls (positive control: ECGM‐2; negative control: 5 × 10^−6^ m Suramin Sodium Salt (TargetMol), prepared in ECGM‐2; untreated control: ECBM‐2, i.e., ECGM‐2 without added growth factors (GF) or FCS; vehicle controls: 8–9 fraction of a blank gradient in equal volume to the maximum volume of EV sample). EV were applied at a dose of 3000 particles per cell, diluted in ECBM‐2. The plate was incubated in a humidified incubator (37 °C, 5% CO_2_) for 8 h. Four replicates per sample were included. Pictures were analyzed using Angiogenesis Analyzer plugin of Fiji software.^[^ ^98^, ^99^ ^]^

### Wound Healing Assay

HUVEC were seeded at 35 000 cells per well onto 48‐well plates coated with 0.1% gelatin (Sigma Aldrich), grown up to confluency, and serum‐starved for 4 h prior to performing the wound. A sterile 200 µL pipette tip was used to create a scratch in the cell monolayer, after which cells were washed twice with DPBS. EV samples were diluted in the proper volume of reduced‐supplementation media [ECGM‐2 minus GF, with 0.5% (v/v) FCS] and added to the appropriate well at a dose of 3000 particles per cell. Positive (fully supplemented ECGM‐2), negative (ECGM‐2 0.5% FCS minus GF), and vehicle (8–9 fraction of a blank gradient in equal volume to the maximum volume of EV sample) controls were prepared. A similar procedure was conducted with the addition of Dynasore in a concentration of 50 × 10^−6^ m. Cells were placed in a humidified environment (37 °C, 5% CO_2_), and images acquired at 0, 8, and 24 h post‐scratch. Wound width was analyzed with using MRI Wound Healing tool of Fiji software.^[^ ^95^, ^98^ ^]^ Percent wound closure was determined relative to time‐point 0 h. Images were acquired using an inverted microscope (DMI6000, Leica Microsystems GmbH).

### HUVEC Proliferation Assay

1 × 10^−6^ m of 5‐ethynyl‐2′‐deoxyuridine (EdU) was added to each well of the wound healing assay. Cells were incubated for 24 h in a humidified incubator (37 °C, 5% CO2), and then fixed with 4% paraformaldehyde for 20 min at RT. The Click‐iT EdU (ThermoFisher Scientific) reaction was performed according to the manufacturer's instructions to reveal EdU positive nuclei. Nuclei were counterstained with DAPI reagent (ThermoFisher Scientific) in a 1:2000 dilution in DPBS. HUVEC were further stained for CD31 marker, as previously described. Fluorescence images were acquired from five randomly selected fields per well using an inverted fluorescence microscope (DMI6000, Leica Microsystems GmbH, Wetzlar, Germany). EdU positive nuclei were presented as a percentage of total DAPI positive nuclei. Nuclei count was performed with Fiji software,^[^ ^98^ ^]^ and encompassed a minimum of 1000 DAPI‐positive nuclei per condition.

### Cardiomyocyte Proliferation Assay

hiPSC‐CM at day 15 of differentiation were seeded at a density of 100 000 cell cm^−2^ in 48‐ and 96‐well plates. 24 h later, cells were treated with EV samples at a dose of 300 particles per cell (time‐point 0 h). 1 × 10^−6^ m of EdU was added to each well. Cells were incubated either for 24 or 48 h in a humidified incubator (37 °C, 5% CO2) and counted by trypan blue exclusion assay. Cells were fixed with 4% paraformaldehyde for 20 min at RT, washed three times with DPBS, permeabilized and blocked with blocking buffer (3% bovine serum albumin, 0.05% Triton X‐100) for 30 min at RT. The Click‐iT EdU (ThermoFisher Scientific) reaction was performed according to the manufacturer's instructions to reveal EdU positive nuclei. For immunostaining, corresponding antibodies were diluted at the desired concentration in blocking buffer [cTnT, 1:200, MS‐295‐PI, Thermo Fisher Scientific; phH3: 1:200, Phospho‐Histone H3 (Ser10) (D7N8E) XP(R) rabbit mAb, 53348S, Cell Signaling Technologies] and incubated with cells overnight at 4 °C. Next day, cells were washed three times with DPBS, and incubated with the appropriate secondary antibody diluted in blocking buffer for 1 h. Nuclei were counterstained with DAPI reagent (ThermoFisher Scientific) in a 1:2000 dilution in DPBS.

### RNA Isolation, Small RNA Sequencing, and Bioinformatics Analysis

Total RNA was isolated from EV samples (*n* = 3 biological replicates corresponding to EV preparations from three independent cultures) using Norgen Biotek Exosomal RNA Isolation Kit (Cat.58000). microRNA library preparation was performed by PCR amplification using Norgen Biotek Small RNA Library Prep Kit (Cat. 63600) and library quality control was achieved using Bioanalyzer to estimate library size and concentration. Libraries were denatured and diluted to the required concentration and then applied onto the suitable flowcell and sequenced using Illumina NextSeq 500 sequencing platforms. FASTQ files were processed using the excerpt small RNA‐seq pipeline version 4.6.2 available on the Genboree Workbench by Norgen Biotek Corp. Mapping and annotation was performed to the reference human genome hg38. miRbase (version 21) was used as reference database for miRNA sequences. Data was filtered and normalized using the trimmed mean of M‐values (TMM) normalization method for differential expression (DE) analysis. DE analysis between each two groups was performed using the EdgeR^[^ ^51^ ^]^ statistical software package and false discovery rate was adjusted through the Benjamini–Hochberg procedure. DE was considered significant for log_2_ fold change ≥ 1 or ≤−1 at *p*‐value and FDR ≤ 0.05.

### miRNA Target Prediction and Pathway Analysis

Target genes for differentially expressed miRNAs were identified using IPA software (Qiagen). A microRNA Target Filter was run on the selected data set, and data filtered by degree of confidence. Only experimentally observed targets were selected. 164 mRNA targets were found. An enrichment analysis based on hypergeometric distribution followed by FDR correction (FDR cutoff of 0.05) was performed on the targeted 164 genes with ShinyGO v0.61,^[^ ^100^ ^]^ for the identification of overrepresented pathways denoted by relevant terms in Kyoto Encyclopedia of Genes and Genomes (KEGG). In parallel, and to corroborate the results obtained with IPA, the same miRNAs with identified targets in IPA were run through DIANA‐miRPath v3.0.^[^ ^59^ ^]^ Overrepresented KEGG pathways were identified using a hypergeometric distribution with FDR correction (FDR cutoff of 0.05).

### Effect of hiPSC‐EV on the PTEN/PI3K/AKT Pathway

HUVEC were seeded at 100 000 cells per well onto six‐well plates. 24 h after seeding, hiPSC‐EV samples were diluted in the proper volume of complete media (ECGM‐2) and added to the appropriate well at a dose of 3000 particles per cell. Untreated (ECGM‐2 only) controls were prepared. Expression of *PTEN* was assessed 24 h post‐treatment by RT‐qPCR, as described above. Primers used for this purpose are available in Table S8 (Supporting Information). Gene expression data were normalized to housekeeping genes *RPLP0* and *GADPH* and relative changes were analyzed using the ∆∆Ct method (treated vs untreated cells). The effect of hiPSC‐EV was additionally confirmed by Western Blot for PTEN (1:1000, #9188, Cell Signaling Technology), total AKT [1:1000, AKT (pan), #4691, Cell Signaling Technology], and phosphorylated AKT [P‐AKT (Ser473), #4060, 1:1000, P‐AKT (Thr308), #13038, Cell Signaling Technology].

### Statistical Analysis

All statistical analysis performed in this paper were done using GraphPad Prism v7. Significance was tested by one‐way ANOVA [*p < 0.05, **p < 0.01, ***p < 0.001, ****p < 0.0001, n.s. (*p* > 0.05)], except where stated. Results are plotted as mean ± SD (*n* = 3 biological replicates, except where stated).

## Conflict of Interest

The authors declare no conflict of interest.

## Supporting information

Supporting Information

## Data Availability

The authors have uploaded small RNA‐seq data to the NCBI's Gene Expression Omnibus (GEO), available by the accession number GSE179323 (accession code available to reviewers upon request), and all relevant data of our EV experiments to the EV‐TRACK knowledgebase (EV‐TRACK ID: EV210151).^[^ ^102^ ^]^
