# Supplementary material for: Bioactivity and miRNome Profiling of Native Extracellular Vesicles in Human Induced Pluripotent Stem Cell‐Cardiomyocyte Differentiation
Source: Adv Sci (Weinh). 2022 Mar 24;9(15):2104296. doi: 10.1002/advs.202104296 (PMC9130911; doi:10.1002/advs.202104296)
Supplement: Supplementary file 1 — Supporting Information [file ADVS-9-2104296-s001.pdf]

ADVANCED SCIENCE
Open Access

Supporting Information

for *Adv. Sci.*, DOI 10.1002/advs.202104296

Bioactivity and miRNome Profiling of Native Extracellular Vesicles in Human Induced Pluripotent Stem Cell-Cardiomyocyte Differentiation

*Ana F. Louro, Marta A. Paiva, Marta R. Oliveira, Katharina A. Kasper, Paula M. Alves, Patrícia Gomes-Alves and Margarida Serra**

Supporting Information

Bioactivity and miRNome Profiling of Native Extracellular Vesicles in Human Induced Pluripotent Stem Cell-Cardiomyocyte Differentiation

Ana F. Louro, Marta A. Paiva, Marta R. Oliveira, Katharina A. Kasper, Paula M. Alves, Patrícia Gomes-Alves, Margarida Serra*

Expanded methods

Gene expression. mRNA was extracted with the High Pure RNA isolation Kit (Roche) ac- cording to the manufacturer's instructions and quantified with Nanodrop 2000c (ThermoFisher Scientific). cDNA synthesis was performed on 50 µg of RNA using the Transcriptor High Fidelity cDNA Synthesis Kit (Roche). Real time quantitative polymerase chain reaction (RT-qPCR) was performed using the LightCycler 480 Instrument II 384-well block (Roche) in the following cycles: incubation at 95 ºC for 10 min; 45 cycles of amplification with denaturation at 95 ºC for 15 sec, and annealing at 60 ºC for 1 min; extension at 72 ºC for 5 min. The primers/probes used for this purpose are available in table 1. The Cycle threshold (Ct) was determined using the LightCycler 480 software (Roche). Gene expression data was normalized to housekeeping genes *RPLP0* and *GADPH* and relative changes were analyzed using the ΔΔCt method (CPC vs. hiPSC, CMm vs. CMi). Primers used for gene expression analysis are summarized in Table S5.

Flow cytometry. Analysis of hiPSC and cardiomyocyte phenotype was performed by flow cytometry (FC). hiPSC were probed for pluripotent stem cell markers (TRA-1-60 and SSEA4) and early differentiation marker (SSEA1) at day 0, while cardiomyocyte markers SIRPa/b, VCAM-1 and cardiac Troponin T (cTnT), were assessed on day 15 and cTnT on day 35 of differentiation. Cell dissociation was either performed with Versene (Gibco®, ThermoFisher Scientific), for hiPSC, or TrypLE Select for cardiomyocytes. Single cells were resuspended and washed twice with FC buffer composed of 5% v/v fetal bovine serum (FBS) in Dulbecco's phosphate buffered saline (DPBS, ThermoFisher Scientific). A minimum of 0.5 million cells were used per sample. For detection of cell-surface epitopes (TRA-1-60, SSEA4 and SSEA1 for hiPSC and SIRPa/b, VCAM-1 for cardiomyocytes), cells were resuspended in the respective primary antibody or isotype control (Table S6) and incubated at 4 ºC for 1 h. For detection of cTnT, an intracellular epitope, cells were fixed and permeabilized using a commercial kit (BD Cytofix/Cytoperm, BD Biosciences), according to the supplier's instructions, after which they were resuspended in primary antibody or isotype control and incubated for 1 h at 4 ºC. When primary unconjugated antibodies were used, an additional incubation with secondary antibody was performed (table 2). At least 20.000 events were recorded per sample on the population of interest. Samples were analyzed on a BD FACS

Celesta (BD Biosciences). Data acquisition was made on BD FACS DIVA software and analysis was performed using FlowJo software (Becton, Dickinson & Company).

Immunofluorescence for cardiomyocyte markers. Aggregates were fixed at room temperature (RT) in 4% paraformaldehyde and 4% sucrose for 20 min and incubated overnight in 30% sucrose prior to OCT embedding. 10 µm sections were sliced and used for staining. Briefly, sections were washed with PBS and blocked in 0.2% FSG, 0.5% Triton X-100 in PBS for 1 h at RT. Slides were incubated overnight at RT with primary antibodies (1:200 dilution, Anti-connexin 43 antibody, C6219, Sigma Aldrich; 1:200 dilution, anti-cardiac Troponin T, MS-295-P1, ThermoFisher Scientific) diluted in 0.125% FSG, 0.1% Triton X-100. The following day slices were washed with PBS and incubated with secondary antibodies for 1 h at RT. Upon secondary antibody removal, cells were washed with PBS and mounted in ProLong Gold Antifade Mountant (Invitrogen™) (Thermo Fisher Scientific). Nuclei were counterstained with DAPI (4',6-Diamidino-2-Phenylindole, Dihydrochloride, ThermoFisher Scientific) in a 1:2,000 dilution in DPBS. Fluorescence images were acquired using an inverted fluorescence microscope (DMI6000, Leica Microsystems GmbH).

miRNA transfection. mirVana miRNA mimics were obtained from Ambion® (ThermoFisher Scientific). miRBase accession numbers and assay IDs of the miRNA mimics employed in this study are included in Table S7. miRNAs were transfected into HUVEC using a standard transfection protocol, at a final concentration of 20 nM. Briefly, 24 h before transfection, 20,000 cells were plated in 250 µL of ECGM-2 in 48-well gelatin-coated plates (0.1% gelatin). On the day of transfection, both transfection reagent (Lipofectamine® RNAiMAX, Life Technologies) and miRNA mimic were diluted in Opti-MEM® (Life Technologies), mixed in a 1:1 ratio, incubated for 10 min at RT, and added to the cells. 24 h after transfection, culture medium was replaced by fresh medium. Wound healing assay was performed on the transfected cells, as previously described. 1 µM of EdU was added to each well of the wound healing assay. Expression of target genes was assessed 24 h and 48 h after transfection by RT-qPCR, as described above. The primers/probes used for this purpose are available in Table S8. Gene expression data was normalized to housekeeping genes *RPLP0* and *GADPH* and relative changes were analyzed using the ΔΔCt method (transfected *vs.* non-transfected cells).

Supplemental Figures

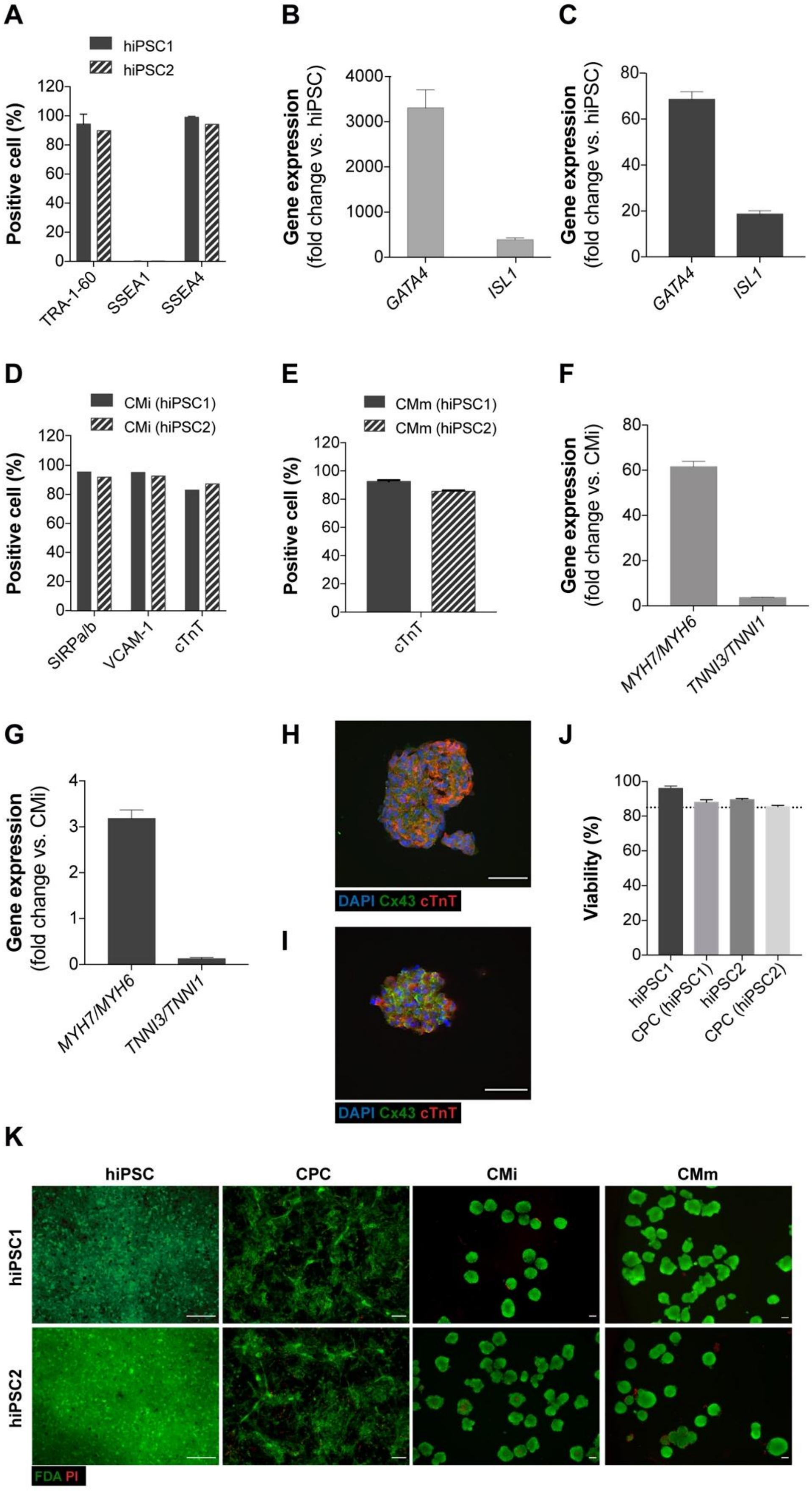

Figure S1. Cell culture characterization. (A) Assessment of pluripotency markers SSEA-4 and TRA-1-60, and early differentiation marker SSEA-1 in hiPSC by flow cytometry. (B) and (C) expression of cardiac progenitor genes *GATA4* and *ISL1* versus hiPSC, for hiPSC1 (B) and hiPSC2 (C). (D) and (E) expression of cardiomyocyte markers SIRPa/b, VCAM-1 and cTnT in CMi (D) and cTnT in CMm (E) by flow cytometry. (F) and (G) *MYH7/MYH6* and *TNNI3/TNNI1* gene expression ratios in CMm versus CMi in hiPSC1 (F) hiPSC2 (G). An increase in both ratios in CMm in comparison to CMi is indicative of cardiomyocyte maturation. (H) and (I) Representative immunofluorescence images of cTnT and Connexin 43 (Cx43) in CMm aggregates obtained from hiPSC1 (H) and hiPSC2 (I). (J) Assessment of cell culture viability by cell counting with Trypan Blue exclusion dye. (K) Representative FDA/PI images of cells at media collection time-points. FDA, shown in green, stains viable cells, while PI, in red, marks non-viable cells. Scale-bar: 100 µm.

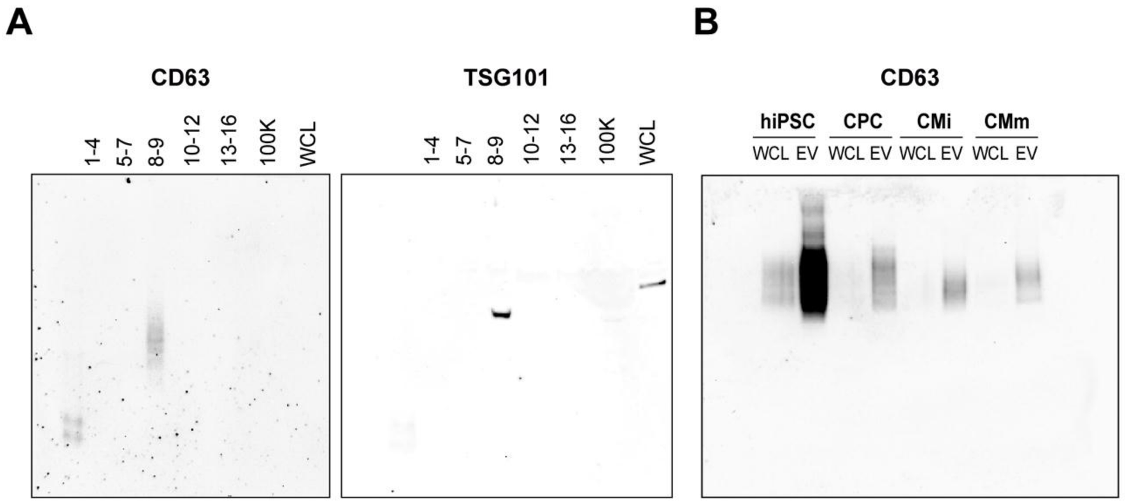

Figure S2. Representative full Western blots featured in Figure 1C (A) and 1G (B). In (A), lanes represent combined fractions of the density gradient, ran along the 100k pellet and the whole cell lysate (WCL). The Western blot shown corresponds to CPC-EV. In (B), EV and WCL are shown alongside for each cell type.

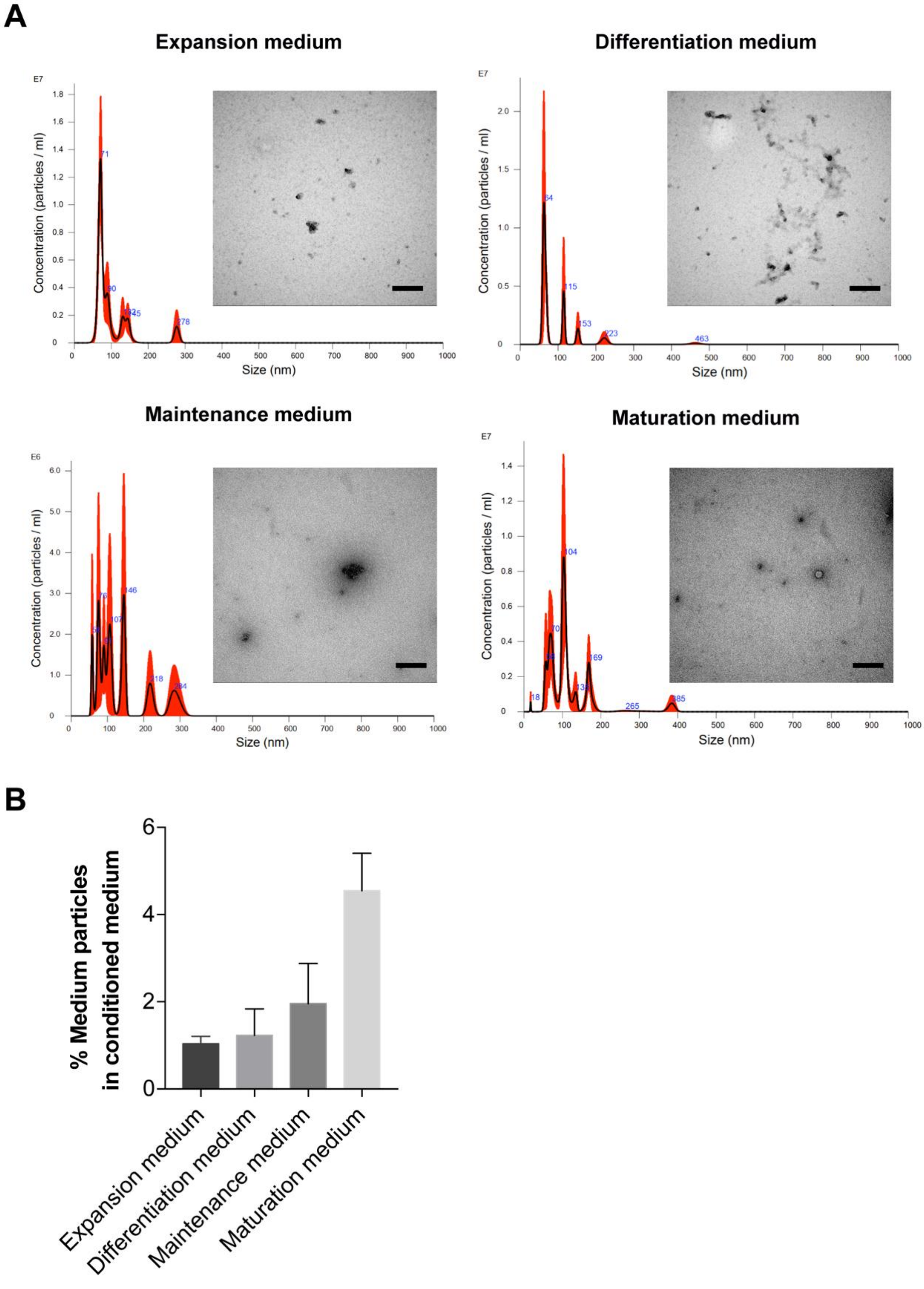

Figure S3. Characterization of background particles present in culture media. (A) Representative size distribution profiles of media samples, processed similarly to conditioned media, analyzed by nanoparticle tracking analysis (NTA) and transmission electron microscopy (TEM). Scale-bars: 200 nm. (B) Particles present in plain culture media as a

percentage of total particles in conditioned media, for each harvest time-point. Data presented as mean ± SD, n=3.

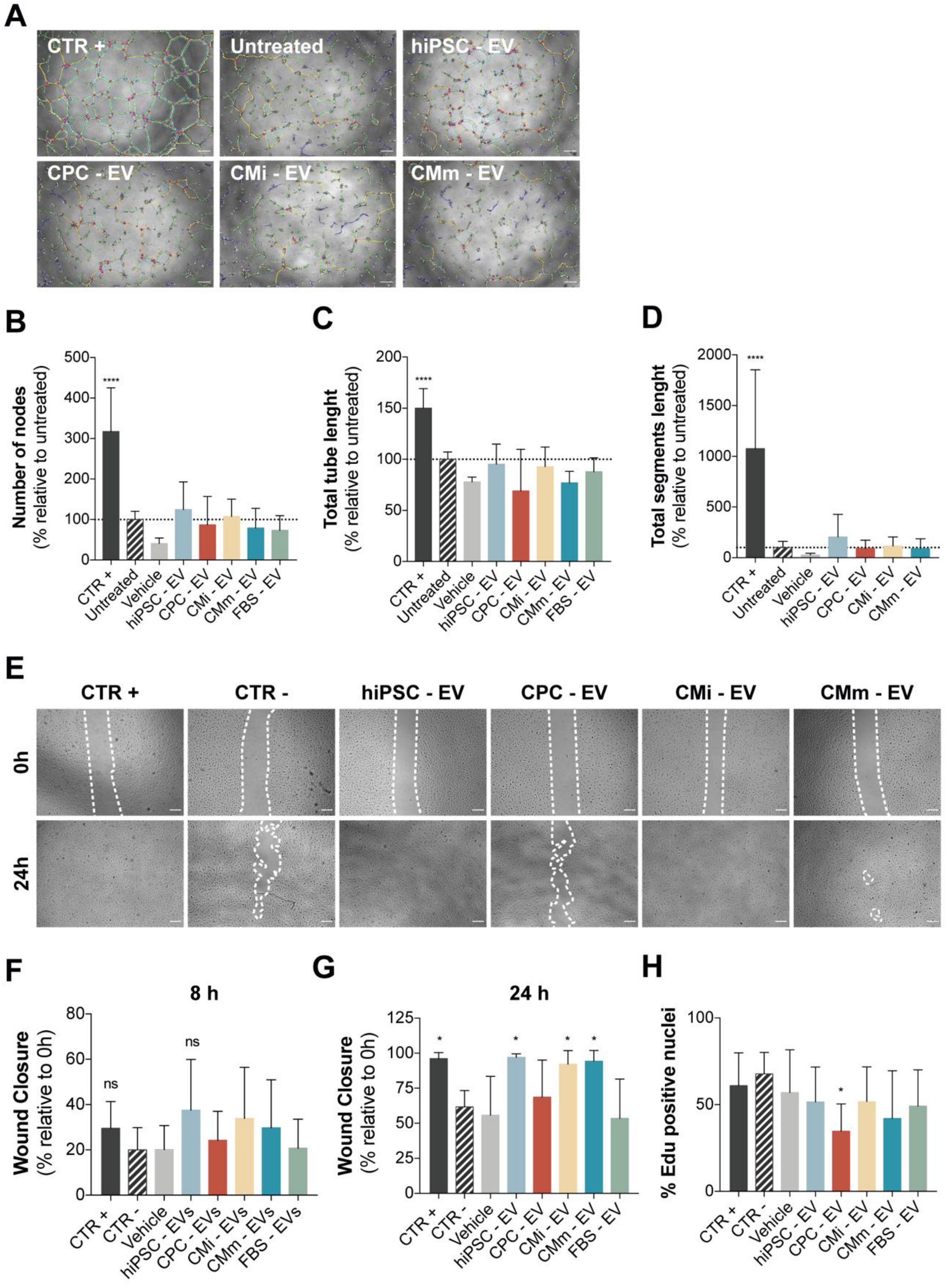

Figure S4. Effect of EV from hiPSC2 on HUVEC. (A) Angiogenic potential of EV samples, evaluated as tube formation at 8 h post-seeding. The number of nodes (pink dots), master junctions (pink circles), master segments (yellow), meshes (light blue), branches (green), and isolated segments (blue) are shown. Scale bar: 100 µm. Tube formation measured as (B) percentage of number of nodes, (C) percentage of total tube length and (D) total tube segments length formed in the assay, relative to the untreated control (taken as 100%). (E) Effect of EV treatment on HUVEC migration evaluated by the wound healing assay. Representative images of cell migration at 0 and 24 h post-scratch. Scale bar: 100 µm. Wound closure at (F) 8 h and G) 24 h post-scratch. (H) HUVEC proliferation in the wound healing assay, assessed by EdU incorporation. Quantification of EdU-positive cells from 5 randomly selected fields per well, equivalent to a minimum of 1000 DAPI-stained nuclei per experiment. n=2. No significant differences were observed for any of the samples. In (B) to (D) and (F) to (K) results are plotted as mean ± SD. Significance was tested against the negative control. *$p < 0.05$** $p<0.01$, **** $p<0.0001$, n.s. ($p>0.05$) by one-way ANOVA with Dunnett's multiple comparisons test, with a single pooled variance. CTR+: positive control, CTR-: negative control.

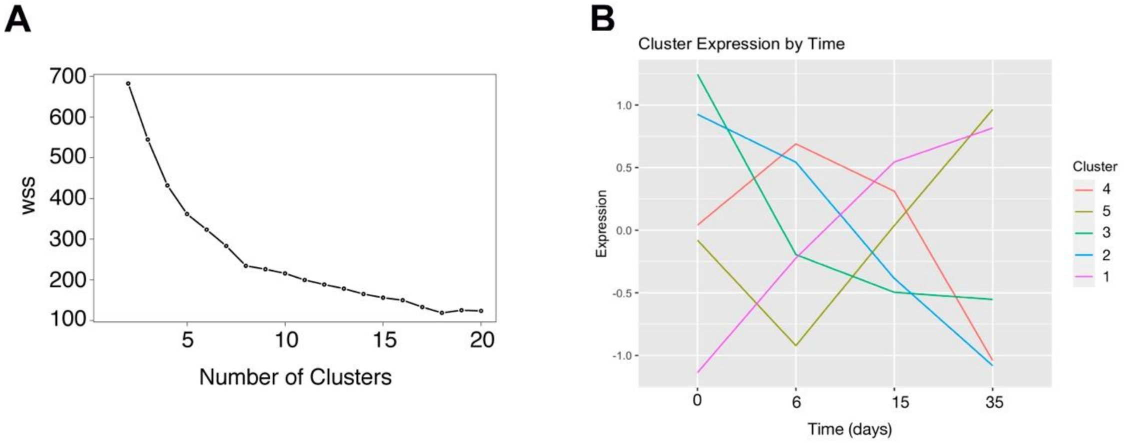

Figure S5. Cluster optimization for Fuzzy c-means clustering. (A) Scree plot illustrating the minimum centroid distance between clusters for a series of cluster numbers. The inflection point of the curve was considered to be x=5 and thus, 5 clusters were chosen as the appropriate k number to perform fuzzy c-means clustering. (B) Assessment of cluster expression by time. Cluster centroids are well separated and present low redundancy.

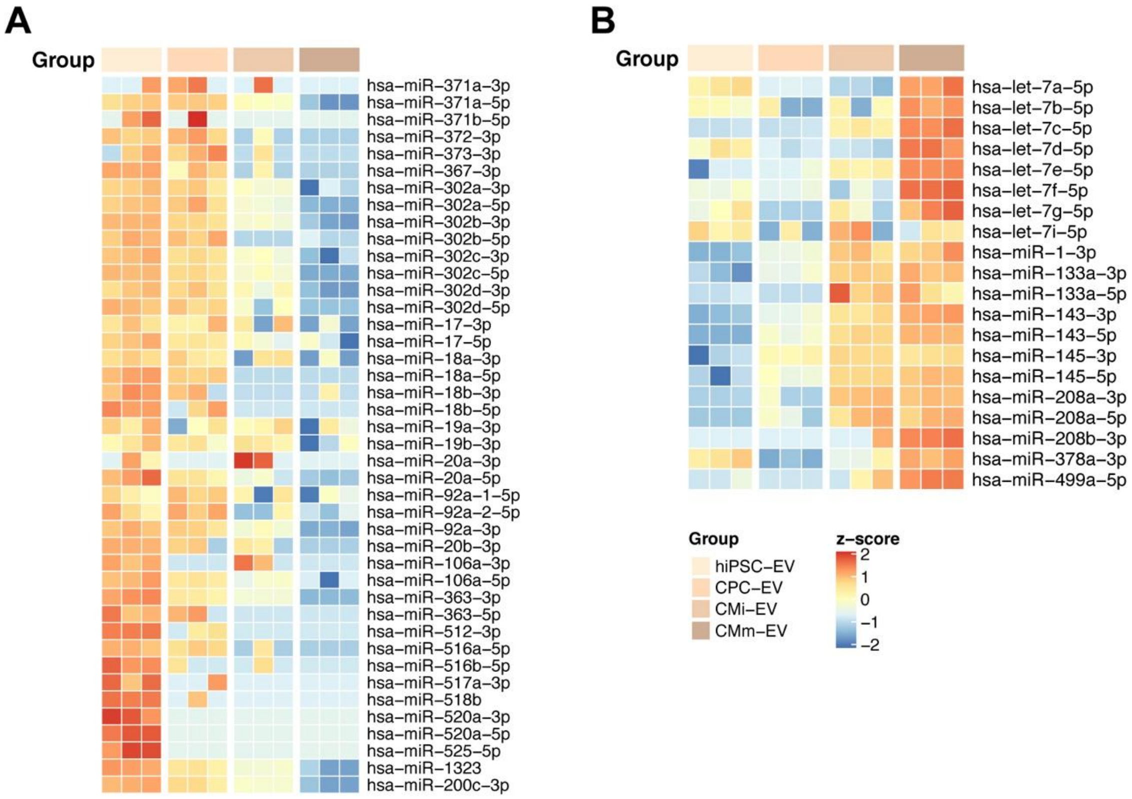

Figure S6. Heatmap representations of the z-score normalized expression levels of (A) stemness- and (B) cardiac specification- associated miRNAs along hiPSC-CM differentiation.

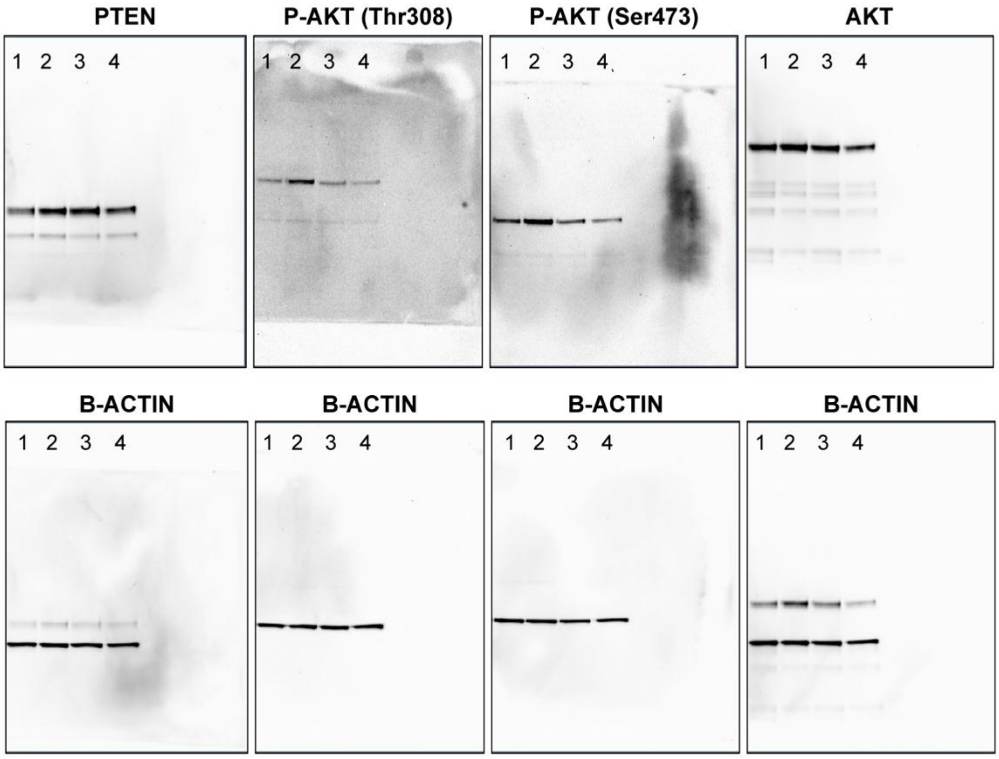

Figure S7. Representative full Western Blots featured in Figure 8 B and C. The numbers on top of each column correspond to the following: 1 = hiPSC-EV treated cells, 2 = cells cultured in 10% FBS (positive control for AKT activation), 3 and 4 = untreated cells (cultured in ECGM-2). The positive control was not included in the analysis but used to assess the ability to activate AKT. Band intensities were always normalized to the individual blot's housekeeping gene (B-ACTIN), shown on the second row of images.

Supplemental Tables

Table S1. Differentially Expressed miRNA between CPC-EV *vs.* hiPSC-EV.

miRNA	Log_2FC	LogCPM	p-value	FDR
hsa-miR-525-5p	-10.592	5.333	1.6E-06	2.0E-05
hsa-miR-135a-2-3p	-10.238	4.982	2.1E-05	2.1E-04
hsa-miR-605-3p	-10.162	4.902	3.3E-05	3.0E-04
hsa-miR-452-5p	-10.119	4.862	5.5E-05	4.9E-04
hsa-miR-3937	-9.683	4.443	1.8E-04	1.5E-03
hsa-miR-105-5p	-9.622	4.369	2.8E-04	2.1E-03
hsa-miR-193b-3p	-9.569	4.338	2.4E-04	1.9E-03
hsa-miR-520a-5p	-9.563	4.330	2.8E-04	2.1E-03
hsa-miR-34b-3p	-9.214	3.968	1.7E-03	1.0E-02
hsa-miR-4326	-9.118	3.884	2.0E-03	1.1E-02
hsa-miR-106a-3p	-9.072	3.848	1.9E-03	1.1E-02
hsa-miR-1251-5p	-8.987	3.766	2.5E-03	1.3E-02
hsa-miR-3660	-8.951	3.723	2.5E-03	1.3E-02
hsa-miR-219a-2-3p	-8.839	3.603	4.0E-03	1.9E-02
hsa-miR-576-5p	-8.803	3.593	3.8E-03	1.9E-02
hsa-miR-671-5p	-8.796	3.577	4.1E-03	2.0E-02
hsa-miR-520a-3p	-8.553	3.346	5.0E-03	2.2E-02
hsa-miR-2467-5p	-8.186	3.032	6.3E-03	2.8E-02
hsa-miR-4664-3p	-7.915	2.752	8.7E-03	3.7E-02
hsa-miR-516b-5p	-4.679	6.526	4.2E-03	2.0E-02
hsa-miR-512-3p	-4.012	7.427	3.8E-04	2.7E-03
hsa-miR-935	-3.464	6.448	6.8E-03	2.9E-02
hsa-miR-135a-5p	-3.328	6.361	1.1E-02	4.6E-02
hsa-miR-146a-5p	-3.195	7.567	7.3E-07	1.1E-05
hsa-miR-221-3p	-2.838	10.640	3.6E-12	1.3E-10
hsa-miR-1298-5p	-2.707	8.569	4.6E-09	8.9E-08
hsa-miR-1323	-2.693	10.231	1.6E-11	4.7E-10
hsa-miR-378a-3p	-2.491	11.208	1.1E-13	5.8E-12
hsa-miR-561-5p	-2.474	6.319	6.0E-03	2.7E-02
hsa-miR-486-5p	-2.452	12.097	1.6E-13	7.6E-12
hsa-let-7a-5p	-2.362	10.039	7.9E-11	2.0E-09
hsa-miR-7-5p	-2.293	8.507	3.0E-06	3.7E-05
hsa-miR-199b-5p	-2.257	7.864	8.3E-04	5.3E-03
hsa-miR-20b-5p	-1.951	9.232	2.5E-03	1.3E-02
hsa-miR-21-5p	-1.902	12.280	3.7E-09	7.4E-08
hsa-miR-222-3p	-1.884	13.298	2.1E-10	4.8E-09
hsa-miR-200c-3p	-1.760	10.402	2.3E-05	2.2E-04
hsa-miR-106a-5p	-1.675	10.576	7.2E-06	8.2E-05
hsa-miR-183-5p	-1.659	14.837	8.6E-07	1.2E-05
hsa-miR-363-3p	-1.641	15.229	4.9E-09	9.0E-08
hsa-miR-93-5p	-1.623	8.957	7.4E-04	4.8E-03
hsa-miR-96-5p	-1.561	9.713	1.0E-04	8.7E-04
hsa-miR-183-3p	-1.532	8.308	1.4E-03	8.7E-03
hsa-miR-20a-5p	-1.513	11.114	6.5E-05	5.7E-04
hsa-miR-182-5p	-1.461	14.642	3.1E-06	3.7E-05

hsa-miR-335-3p	-1.425	12.357	1.2E-06	1.6E-05
hsa-miR-221-5p	-1.403	10.451	1.3E-05	1.4E-04
hsa-miR-577	-1.381	11.440	2.8E-04	2.1E-03
hsa-miR-204-5p	-1.256	9.866	3.3E-04	2.4E-03
hsa-miR-302c-3p	-1.213	11.919	3.3E-04	2.4E-03
hsa-miR-302c-5p	-1.191	10.474	4.8E-04	3.3E-03
hsa-miR-302b-3p	-1.132	17.082	1.2E-05	1.3E-04
hsa-miR-340-5p	-1.076	9.104	6.8E-03	2.9E-02
hsa-miR-17-5p	-1.028	9.542	4.9E-03	2.2E-02
hsa-miR-103a-3p	-1.025	10.218	2.2E-03	1.2E-02
hsa-miR-192-5p	1.028	11.375	1.9E-03	1.1E-02
hsa-miR-423-5p	1.126	13.727	1.6E-03	9.7E-03
hsa-miR-200b-3p	1.330	12.269	2.8E-05	2.6E-04
hsa-miR-484	1.342	12.093	1.5E-05	1.5E-04
hsa-miR-24-3p	1.418	8.855	4.3E-04	2.9E-03
hsa-miR-23b-3p	1.480	9.242	1.0E-03	6.5E-03
hsa-miR-501-3p	1.859	12.025	8.0E-07	1.2E-05
hsa-miR-500a-3p	1.908	10.705	1.6E-09	3.5E-08
hsa-miR-146b-5p	1.917	12.261	5.0E-08	8.4E-07
hsa-miR-7977	1.996	7.147	3.9E-03	1.9E-02
hsa-miR-99b-5p	1.998	15.622	3.2E-12	1.2E-10
hsa-miR-125b-5p	2.081	9.788	1.2E-06	1.6E-05
hsa-miR-502-3p	2.100	7.465	1.2E-03	7.6E-03
hsa-miR-125a-5p	2.107	14.277	7.9E-12	2.6E-10
hsa-miR-184	2.372	7.129	3.5E-03	1.8E-02
hsa-miR-133a-3p	3.483	6.638	1.2E-03	7.6E-03
hsa-miR-143-3p	3.679	11.115	1.2E-16	8.0E-15
hsa-miR-1269a	4.148	4.932	1.0E-02	4.3E-02
hsa-miR-125b-2-3p	4.332	6.219	6.4E-04	4.3E-03
hsa-miR-3605-3p	4.579	5.139	3.8E-03	1.9E-02
hsa-miR-1-3p	4.807	9.390	2.6E-22	2.5E-20
hsa-miR-23b-5p	4.960	5.711	2.5E-04	2.0E-03
hsa-miR-1247-5p	5.152	6.280	5.9E-06	6.9E-05
hsa-miR-145-3p	5.510	10.079	7.8E-08	1.3E-06
hsa-miR-99a-5p	5.579	9.847	3.3E-22	2.6E-20
hsa-miR-10a-5p	5.948	8.834	2.4E-08	4.3E-07
hsa-miR-375-3p	6.288	11.470	1.8E-32	3.5E-30
hsa-miR-654-3p	7.067	6.825	8.5E-11	2.1E-09
hsa-miR-483-5p	7.367	7.442	4.5E-11	1.2E-09
hsa-miR-483-3p	7.378	3.785	5.0E-03	2.2E-02
hsa-miR-370-3p	7.783	5.328	1.3E-05	1.4E-04
hsa-miR-127-3p	8.945	6.897	1.2E-12	5.3E-11
hsa-miR-143-5p	9.573	4.347	2.6E-04	2.0E-03
hsa-miR-122-5p	10.384	8.946	1.3E-39	4.9E-37
hsa-miR-1263	12.843	7.562	3.5E-25	4.5E-23

Table S2. Differentially Expressed miRNA between CMi-EV *vs.* CPC-EV.

miRNA	Log_2FC	LogCPM	p-value	FDR
hsa-miR-302b-5p	-11.042	6.084	5.07E-08	7.31E-07
hsa-miR-489-3p	-10.600	5.651	3.37E-06	3.53E-05
hsa-miR-18a-5p	-10.279	5.338	2.82E-05	2.32E-04
hsa-miR-9-3p	-10.192	5.253	5.58E-05	4.23E-04
hsa-miR-370-3p	-10.159	5.221	7.01E-05	5.16E-04
hsa-miR-654-3p	-9.435	6.713	2.32E-08	3.49E-07
hsa-miR-1908-3p	-8.898	4.015	1.11E-02	4.70E-02
hsa-miR-127-3p	-6.094	6.823	3.30E-04	1.87E-03
hsa-miR-372-3p	-5.538	7.393	8.19E-04	4.29E-03
hsa-miR-200b-3p	-5.210	11.719	4.15E-29	4.79E-27
hsa-miR-577	-4.964	9.527	5.91E-06	5.68E-05
hsa-miR-302d-5p	-4.467	8.116	2.52E-04	1.48E-03
hsa-miR-302a-5p	-4.278	16.351	1.63E-19	8.05E-18
hsa-miR-302b-3p	-3.733	15.403	3.44E-25	2.98E-23
hsa-miR-371a-5p	-3.513	9.601	3.86E-18	1.48E-16
hsa-miR-302c-5p	-3.509	8.776	7.38E-12	1.70E-10
hsa-miR-20b-5p	-3.371	7.008	1.15E-02	4.80E-02
hsa-miR-320d	-3.255	7.863	9.01E-05	5.99E-04
hsa-miR-200c-3p	-3.241	8.321	5.85E-06	5.68E-05
hsa-miR-302c-3p	-3.176	10.231	4.95E-12	1.22E-10
hsa-miR-222-3p	-3.170	11.110	3.35E-15	8.93E-14
hsa-miR-375-3p	-3.164	11.497	2.54E-11	5.49E-10
hsa-miR-183-5p	-3.079	12.831	1.32E-16	4.15E-15
hsa-miR-182-5p	-3.071	12.785	2.37E-16	6.82E-15
hsa-miR-96-5p	-2.984	7.787	1.56E-04	9.66E-04
hsa-miR-1323	-2.842	7.423	1.04E-03	5.39E-03
hsa-miR-302d-3p	-2.824	8.771	8.83E-06	8.25E-05
hsa-miR-302a-3p	-2.819	9.290	1.12E-08	1.75E-07
hsa-miR-25-5p	-2.802	7.984	4.90E-04	2.69E-03
hsa-miR-106a-5p	-2.422	8.645	1.77E-05	1.53E-04
hsa-miR-205-5p	-2.323	10.136	1.54E-09	2.81E-08
hsa-miR-221-5p	-1.994	8.798	2.34E-06	2.53E-05
hsa-miR-320c	-1.750	8.588	1.74E-03	8.75E-03
hsa-miR-320b	-1.717	9.241	7.75E-05	5.48E-04
hsa-miR-146b-5p	-1.657	12.211	1.97E-06	2.28E-05
hsa-miR-20a-5p	-1.611	9.470	2.73E-04	1.58E-03
hsa-miR-204-5p	-1.482	8.437	8.27E-03	3.67E-02
hsa-miR-363-3p	-1.274	13.572	1.20E-04	7.73E-04
hsa-miR-92b-3p	-1.246	14.326	1.75E-04	1.06E-03
hsa-miR-335-3p	-1.157	10.895	3.58E-03	1.70E-02
hsa-miR-224-5p	-1.135	9.920	2.49E-03	1.21E-02
hsa-miR-24-3p	1.061	9.899	2.85E-03	1.37E-02
hsa-miR-23a-3p	1.111	8.827	9.51E-03	4.15E-02
hsa-miR-30e-5p	1.155	13.609	3.99E-04	2.23E-03
hsa-miR-1271-5p	1.224	9.946	3.66E-03	1.70E-02
hsa-miR-30c-5p	1.270	13.604	1.23E-04	7.73E-04
hsa-miR-125a-5p	1.280	15.623	3.20E-05	2.57E-04

hsa-miR-30b-5p	1.556	9.497	2.26E-05	1.91E-04
hsa-miR-378a-3p	1.594	10.370	5.62E-05	4.23E-04
hsa-miR-30d-5p	1.599	14.067	5.24E-07	6.72E-06
hsa-miR-21-5p	1.662	11.981	5.65E-06	5.68E-05
hsa-miR-1301-3p	1.729	9.146	8.57E-05	5.82E-04
hsa-miR-22-3p	1.903	11.024	1.78E-06	2.12E-05
hsa-miR-30a-5p	1.907	12.046	8.36E-09	1.38E-07
hsa-miR-483-5p	2.171	9.755	1.00E-02	4.28E-02
hsa-miR-145-3p	2.173	12.376	2.00E-09	3.46E-08
hsa-miR-145-5p	2.282	8.619	3.29E-05	2.58E-04
hsa-miR-125b-2-3p	2.378	8.635	1.52E-05	1.36E-04
hsa-let-7e-5p	3.483	8.334	4.89E-07	6.51E-06
hsa-miR-10a-5p	3.623	12.406	9.64E-11	1.96E-09
hsa-miR-181a-2-3p	3.756	8.590	5.65E-08	7.81E-07
hsa-miR-143-3p	3.790	14.756	1.06E-17	3.68E-16
hsa-miR-143-5p	4.175	8.409	1.17E-09	2.25E-08
hsa-miR-133a-3p	4.268	10.694	2.57E-19	1.11E-17
hsa-miR-125b-1-3p	4.665	7.854	2.37E-04	1.41E-03
hsa-miR-181a-5p	4.699	8.254	7.76E-05	5.48E-04
hsa-miR-99a-5p	4.812	14.527	1.49E-19	8.05E-18
hsa-miR-4662a-5p	5.072	6.954	3.68E-03	1.70E-02
hsa-miR-208a-5p	5.314	7.224	1.22E-04	7.73E-04
hsa-miR-125b-5p	5.359	14.730	1.44E-29	2.50E-27
hsa-miR-490-3p	5.539	8.688	1.65E-06	2.04E-05
hsa-miR-1-3p	5.589	14.819	1.34E-24	9.26E-23
hsa-miR-133b	6.271	5.247	6.22E-04	3.31E-03
hsa-miR-100-5p	6.688	17.025	1.63E-59	5.63E-57
hsa-miR-208a-3p	6.691	8.659	2.20E-06	2.46E-05
hsa-let-7c-5p	8.163	5.320	7.92E-05	5.48E-04
hsa-miR-887-3p	8.961	4.060	9.59E-03	4.15E-02
hsa-miR-671-5p	9.226	4.309	7.56E-03	3.40E-02
hsa-miR-133a-5p	9.992	5.051	5.39E-04	2.91E-03
hsa-miR-490-5p	10.324	5.375	1.54E-05	1.36E-04

Table S3. Differentially Expressed miRNAs between CMm-EV *vs*. CMi-EV.

miRNA	Log_2FC	LogCPM	p-value	FDR
hsa-miR-302c-5p	-10.011	4.681	1.07E-04	1.47E-03
hsa-miR-302d-3p	-8.501	5.280	1.74E-05	2.78E-04
hsa-miR-371a-5p	-7.768	5.499	4.54E-06	8.60E-05
hsa-miR-200c-3p	-7.471	4.488	3.13E-03	2.59E-02
hsa-miR-1270	-7.087	5.085	4.91E-04	5.73E-03
hsa-miR-205-5p	-6.027	7.075	5.60E-06	9.98E-05
hsa-miR-375-3p	-5.422	7.723	3.23E-07	6.52E-06
hsa-miR-302b-3p	-5.134	11.106	1.04E-17	5.23E-16
hsa-miR-200b-3p	-4.918	6.035	4.58E-04	5.55E-03
hsa-miR-182-5p	-4.807	9.103	2.14E-20	1.29E-18
hsa-miR-302c-3p	-4.642	6.472	1.38E-03	1.31E-02
hsa-miR-1263	-4.578	5.913	1.45E-03	1.33E-02
hsa-miR-483-5p	-4.461	8.986	1.61E-03	1.44E-02
hsa-miR-302a-5p	-4.293	11.575	4.23E-28	6.41E-26
hsa-miR-183-5p	-4.146	9.171	4.60E-15	1.74E-13
hsa-miR-363-3p	-2.403	11.544	4.63E-14	1.56E-12
hsa-miR-130b-5p	-2.178	10.933	2.21E-09	6.09E-08
hsa-miR-335-3p	-2.100	8.990	1.36E-04	1.72E-03
hsa-miR-92a-3p	-1.599	14.646	1.03E-07	2.22E-06
hsa-miR-148a-3p	-1.360	12.826	1.83E-05	2.78E-04
hsa-miR-501-3p	-1.357	12.103	1.29E-03	1.26E-02
hsa-miR-500a-3p	-1.313	10.426	1.26E-03	1.26E-02
hsa-miR-148b-3p	-1.299	9.372	7.18E-04	7.51E-03
hsa-miR-143-5p	1.070	9.453	3.16E-03	2.59E-02
hsa-miR-378a-3p	1.519	11.392	7.78E-05	1.12E-03
hsa-miR-125a-5p	1.715	16.716	1.42E-08	3.59E-07
hsa-miR-143-3p	1.846	16.341	2.49E-08	5.81E-07
hsa-miR-181b-5p	2.037	9.026	1.28E-04	1.69E-03
hsa-miR-181a-5p	3.718	11.502	3.88E-17	1.68E-15
hsa-let-7e-5p	4.181	11.957	3.82E-30	1.16E-27
hsa-let-7f-5p	4.368	8.845	3.44E-10	1.04E-08
hsa-let-7a-5p	4.894	10.853	7.66E-27	7.73E-25
hsa-miR-346	5.164	8.494	1.70E-05	2.78E-04
hsa-let-7c-5p	5.167	9.989	3.35E-22	2.53E-20
hsa-let-7d-5p	5.629	4.842	7.05E-04	7.51E-03
hsa-miR-508-3p	9.863	4.523	7.05E-04	7.51E-03

Table S4. miRNA and corresponding mRNA targets in the PI3K/AKT pathway identified by IPA®

miRNA	mRNA targets
hsa-miR-183-5p	*FOXO1*
hsa-miR-200c-3p	*PTEN*
hsa-miR-302c-3p	*CDKN1A*
hsa-miR-335-3p	*ITGA5, ITGB3*
hsa-miR-363-3p	*PTEN*

Table S5. List of primers used in RT-qPCR characterization of hiPSC-CM.

Gene	Reference	Gene	Reference
GADPH	Hs99999905	***MYH6***	Hs01101425
RPL0	Hs99999902	***MYH7***	Hs01110632
GATA4	Hs00171403	***TNNI1***	Hs00913333
ISL1	Hs00158126	***TNNI3***	Hs00165957

Table S6. Antibodies for flow cytometry.

**Extracellular epitopes**			
**Antibody**	**Antibody type**	**Dilution**	**Reference**
Anti-SSEA4, FITC (IgG3)	Primary conjugated	1:10	560126 (BD Biosciences)
Isotype control IgG3, FITC	Primary conjugated	2:5	355578 (BD Biosciences)
Anti-SSEA1, FITC (IgM)	Primary conjugated	1:10	sc-21702 (Santa Cruz Biotechnology)
Isotype control IgM, FITC	Primary conjugated	1:400	553474 (BD Biosciences)
Anti-TRA-1-60 (IgM)	Primary unconjugated	1:10	sc-21705 (Santa Cruz Biotechnology)
Isotype control IgM	Primary unconjugated	1:10	sc-3881 (Santa Cruz Biotechnology)
Anti-SIRPa/b, PE (IgG1k)	Primary conjugated	1:20	323806 (BioLegend)
Anti-CD106 (VCAM-1), IgG1k	Primary conjugated	1:5	555647(BD Pharmigen)
Isotype control IgG1k, PE	Primary conjugated	1:5	555749 (BD Pharmigen)

**Intracellular epitopes**			
**Antibody**	**Antibody type**	**Dilution**	**Reference**
Anti-Cardiac Troponin T (IgG1)	Primary unconjugated	1:200	MS-295-PI (Thermo Fisher Scientific)
Isotype control IgG1	Primary unconjugated	2:5	sc-3877 (Santa Cruz Biotechnology)

Table S7. miRNA mimics miRbase accession numbers and assay ID.

miRNA	miRBase Accession	Assay ID
hsa-miR-183-5p	MIMAT0000261	MC12830
hsa-miR-200c-3p	MIMAT0000617	MC11714
hsa-miR-302c-3p	MIMAT0000717	MC10571
hsa-miR-335-3p	MIMAT0004703	MC13018
hsa-miR-363-3p	MIMAT0000707	MC10149

Table S8. List of primers used in RT-qPCR after transfection and for evaluation of the effect of hiPSC-EV on the PI3K/AKT pathway.

Gene	Reference	Gene	Reference
GADPH	Hs99999905	***CDKN1A***	Hs00355782_m1
RPL0	Hs99999902	***CCND1***	Hs00765553_m1
PTEN	Hs02621230_s1	***TWF1***	Hs00702289_s1
